# Intersections and Divergences Between Empathizing and Mentalizing: Development, Recent Advancements by Neuroimaging and the Future of Animal Modeling

**DOI:** 10.3389/fnbeh.2019.00212

**Published:** 2019-09-13

**Authors:** Luca Cerniglia, Letizia Bartolomeo, Micaela Capobianco, Sara Lucia M. Lo Russo, Fabiana Festucci, Renata Tambelli, Walter Adriani, Silvia Cimino

**Affiliations:** ^1^Faculty of Psychology, International Telematic University Uninettuno, Rome, Italy; ^2^Center for Behavioral Sciences and Mental Health, Istituto Superiore di Sanità (ISS), Rome, Italy; ^3^Department of Dynamic and Clinical Psychology, “Sapienza” University of Rome, Rome, Italy

**Keywords:** functional imaging, neural correlates, theory of mind, metacognition, social interactions, prosocial behavior, reciprocity

## Abstract

Both mentalization and empathy allow humans to understand others, through the representation of their mental states or their mood, respectively. The present review aims to explain those characteristics which are shared between empathy and the Theory of Mind. Research in neuroscience, based on naturalistic paradigms, has shown that abilities to mentalize and to empathize are associated with the activation of different neuro-cognitive circuits. As far as mirror-neuron processes are concerned, some structures (like Anterior Insula, AI; Anterior Cingulate Cortex, ACC) play a role both in the representation of one’s own affective states and in comprehension of the same affective state when experienced by others. As for mentalization, the temporal parietal junction (TPj) and temporal poles (TP), the upper posterior temporal sulcus (pSTS) and the medial prefrontal cortex (mPFC) are greatly involved: the latter appears involved in the attribution of one’s own and others’ temperaments. Interestingly, the ventral/orbital portion of the PFC (orbito-frontal cortex, OFC) is involved in subserving shared affective experience during cognitive mentalizing. This brain region represents a point of overlap, from a psycho-biological point of view, where emotional mirroring and affective cognition meet up. As for animal models, laboratory rodents can well be tested for prosocial behavior. Some examples include deliberate actions, allowing another conspecific the possibility to feed (“giving food”): this willingness can vary across donors, depending on how the recipient is perceived. Other examples include the possibility to let a trapped conspecific come out (“giving help”). The state-of-the-art knowledge about this theme can inform the programming of specific clinical interventions, based on the reinforcement of empathic and/or mentalization abilities.

Human ability to adapt and survive is largely based on interpersonal skills, in particular on the ability to understand and to cooperate with others. To preserve this precious capacity, social cognition relies on many mechanisms: among these, empathy and Theory of Mind are the most important ones. Although both Theory of Mind and empathy allow human beings to understand others through the representation of their mental or of their emotional states, respectively, research in the field of neuroscience has shown that the abilities of mentalizing and empathizing are associated with the activation of different neuro-cognitive circuits. On the other hand, recent studies based on naturalistic paradigms have shown that the neural systems involved in sharing and mentalization experiences play complementary roles during complex social interactions.

## Method

The present review has the purpose to discuss some features, differences and overlaps in the concepts used to define empathy and Theory of Mind: this issue will be addressed both from a psychological and a neuro-biological point of view. We chose to prepare a narrative review (Green et al., 2006; Pan, 2008) because it is an interpretative-qualitative form of accounting about research (Ferrari, 2015): it allows synthesizing the findings of literature about a given topic and expand our knowledge about it (Grant and Booth, 2009).

The methodological approach (Egger et al., 2001) consisted in the collection of articles available in the international literature through electronic databases such as PROQUEST, PSICARTICLES, and PSYCINFO, published from 1995 until 2018. We used Scopus to verify the scientific relevance of the journals where these documents were published (as indicated in Montoya et al., 2018). The literature search has been carried out using various combinations of the following keywords, for example: “theory of mind,” “mentalizing,” “role-taking,” “perspective taking,” “emotional contagion” and “empathic concern.”

The titles and abstracts of each identified article have been examined and a subset of them has been selected based on relevance: the latter was judged in terms: (1) of the number of citations gained in other articles of the same field; and (2) of the impact-factor of journals in which they have been published. Other criteria of inclusion have been: articles in Italian or English language, published in peer-reviewed journals, with the exclusion of any University Degree Thesis. At the end of the process, a total of 98 scientific articles have been included.

Another purpose of the present review was to address the issue of animal modeling. We run a PubMed search with exactly the same keywords as above, with the addition of “rat” or “animal model” or “preclinical” and some review articles were returned, supporting the idea that empathy-like phenomena, which are not limited to humans but widespread across mammals, also can be studied in laboratory setting. Further support to such notion can be found elsewhere (Mogil, 2012; Panksepp and Panksepp, 2013; Keum and Shin, 2016). Of course, it is not easy to state that laboratory rodents can “feel” a conspecific as a “feeling being.” To deliberate whether this may happen, and to what extent, is perhaps a matter of philosophy and is beyond our current purposes. We decided to limit our literature search to those test paradigms in which the following criteria are met: (1) the tested experimental subjects are more than just one; and (2) the behavior observed is not just a reaction to spontaneous behavior expressed by (an)other subject(s).

The second criterion was intended to exclude all classical social interactions. Many tests are performed with laboratory rodents to measure e.g., affiliative/playful profiles, aggressive/competitive traits, sexual attraction and approach. However, in all these cases, the behavior shown by one subject does not require this subject to perceive other subjects as “agents that feel.” Or, alternatively, it is not essential for the one subject to capture the feeling felt by the other subject. In other words, many social behaviors in animals can well be expressed as an instinct or occurring *via* automatic routines. We rather sought to focus on those (more complex) behaviors where a focal subject, in order to foresee which next action to perform in favor of the conspecific, needs to grasp and interpreter its mood; alternatively, the reaction of the focal subject is depending on perceiving the conspecific’s action as “deliberate” or “causal.”

The above reasoning implies that phenomena of “contagion” are beyond the present purposes. There are quite simple settings where a demonstrator is placed when suffering from social stress or pain, and the observing subjects are evaluated. Usually, the latter quickly begin to show a response that is similar to the observed one (see e.g., Carnevali et al., 2017; Laviola et al., 2017). These situations resemble the vicarious experiences of pain or emotions described in humans in paragraph 3.1; yet, it can be questioned whether this represents a real form of metacognition. The fact that a rodent becomes more prone to stress or pain, when observing another conspecific already showing such signs, does not imply that the subject is recognizing the other as a “subject that can feel stress or pain.” We are aware of the limitations related to the work with rodents: all internal states can only be inferred from what animal subjects do. A logical consequence of this assumption is that we can explore what rodents do in favor of each other, in search of abilities reminescent of mentalization, adopting protocols similar to those described for humans. In paragraph 3.2, we are interested in paradigms whereby animal subjects can perform actions in favor of (an)other conspecific(s). These actors—given the situation—are then recognized as “agents” who may either promote a helpful action or benefit from that action. According to these premises, test paradigms are only taken into account if they allow situations such as “helping” or “donating.” The asymmetry is implicit, in that one subject will act as a “donor” while other(s) will simply be the recipient(s). Yet, from the side of the donor, the willingness to donate may be modulated by the “mood” (i.e., the inner state) of the recipient, or at least by the “empathic” perception (of the recipient by the donor): some recipients may be eliciting more (or less) donations, in the same donor, meaning that empathy is involved.

On the other hand, rodents are also capable of a range of higher-order social skills, like cooperation and reciprocity: these imply that the “other” is somewhat perceived as “someone” that can act prosocially (or not) and perform (or not) actions to reciprocal benefit. It is assumed that such a reciprocal kind of prosocial behavior is reinforced only if benefit to *another* subject is perceived as still rewarding by the *one* subject.

## Theoretical Framework and Constructs

Despite being an important concept for social life, it is not easy to define the word “empathy.” This term has been used by philosophers, sociologists, ethologists, psychologists and neuroscientists in different ways. The greek etymology is from the verb “en-pathein” that means “feeling from inside.” In the common language, the meaning of empathy is associated to the capacity of sharing emotional states with other people: this involves the ability of getting into their point of view and understanding their own way to evaluate things and situations.

In his book “Zero degrees of empathy,” Baron-Cohen (2011, p. 11) defines empathy as “the ability to understand what the other one is thinking or feeling, and to react to his/her thoughts and feelings with the appropriate feeling and—hence—deliberation. This definition includes the correct recognition of emotional and mental states of the other, as well as reacting with the consequent motivational drive.

A central nucleus of the debate concerning the structure of empathy addresses, more specifically, the investigation of all components that are necessary for its definition. The research on empathy has been divided for very long time in two different perspectives: one that considers empathy as a purely affective experience (affective empathy), and another one that considers empathy in its cognitive nature (cognitive empathy). The theories that give priority to the affective aspects in defining empathy were mainly shared by social and clinical psychologist, and represented the only possible approach until the middle years of the last century.

Psychoanalysts and psychotherapists (Rogers, 1959; Kohut, 1984) share a perspective according to which empathy can be described like a semi-voluntary process of emotive activation, while for other authors it is innate: this process involves the sharing of emotional experiences by others. According to this approach, to empathize with someone means: (1) to share the emotions that the other one lives; or (2) experiencing the same emotion, although vicariously. Such a position gives importance to the affective aspects of empathy.

In the social field, the empathic experience has been defined by Allport (1937) as an immediate sharing of the mental state of the other, or an “immediate sympathetic response,” followed by instinctively assuming the posture and the facial expression observed in the other. An important contribution, useful to study the conscious mechanisms of empathic sharing, came from Mead and Mind (1934). He defined empathy as the ability to “get in the role of the other.” Mead’s definition has been a fundamental pillar for all authors that, starting from the 50s–60s, decided to study the cognitive process of thought and empathy.

Starting from the 60s, the word “empathy” has gained a mainly completely cognitive meaning: authors have turned the object of their studies into the role of cognition and of thought in the empathic responses. Even previous studies (see Dymond, 1949) introduced the term of “role-taking” to indicate the comprehension of another’s point of view. When used in this way, the term “empathy” can be considered as a synonymous of the “assumption of role” or of “perspective taking.” Following this line of thought, Hogan (1969) defined empathy as the ability of understanding the condition and the mental state and the subjective experience of others. In this formulation, empathy is a purely cognitive skill that does not require feeling (simultaneously) others’ emotional states.

This specific vision of empathy is shared by the theory about metacognition of mind, as defined by Gallagher and Frith (2003, p. 77): this concept refers to the ability to attribute mental states (such as beliefs, emotions, desires, intentions) to ourselves and to others as well as to forecast, based on these inferences, future behavior in ourselves and by others. From the 80-s, both the affective and the cognitive components of empathy, have been considered as co-occurring to generate an empathic response. Authors have started to talk of multi-factorial models of empathy.

According to the just-mentioned visions, empathy phenomena would only be possible thanks to the integrated action of both affective and cognitive components. The latter intervenes and contributes at very different levels, also depending on the complexity of the situation and on the level of analysis operated by the subject. This multi-factorial concept comprises different levels of “sharing,” from the primitive and automatic forms (similar to emotional contagion) to the most evolved forms.

According to Feshbach (1983), empathy consists of three elements: the ability of discriminate and correctly recognize the emotions expressed by the other; the ability to assume the perspective and the role of the other; self-modulation, by adhering to the necessities of the other, following a “moral” directive gained through empathy. Hoffman (1987, p. 479), one of the most important authors in this field, has defined empathy as an affective response that is “most appropriate to the situation of another person than to own one.” Feeling empathy does not mean to experience exactly what the other lives, but to understand and/or to share in a vicarious way the emotions and needs he feels. According to such multi-factorial models, empathy is defined as a process that involves and integrates cognitive and affective components; in fact, it requires the comprehension of the mental and emotional state of the other as well as the ability to respond in the most adaptive way.

Starting from the work of Hoffman (1987) and Batson et al. (1991), “empathy” has been defined as “an affective response that is identical or very similar to what the other person experiences or is about to experience in a specific context” and “a response that is triggered by the understanding of the mental state of another person.” To capture these multi-faceted concept, Davis (1980) proposes a multi-dimensional definition of empathy and a new tool for its measure, termed IRI (Interpersonal Reactivity Index).

In order to realize an integration between cognitive and emotional factors within a single definition, we can cite the definition of empathy formulated by Bischof-Köhler (1991). For this author, empathy is “the ability of understanding others through the direct sharing and immediate experience of their affective state, where the subject that empathizes keeps the consciousness that his current affective state is indeed mirroring the affective state of the other.” This definition entails a couple of fundamental advantages: on the one side, it allows differentiating empathy from most primitive forms of emotional contagion; on the other hand, it allows distinguishing the “true” empathy from the more cognitive psychological processes for understanding others (ranging from perspective taking to the Theory of Mind).

Similarly, according to Ickes (1997), empathy is a multi-dimensional construct: it can be defined as a complex form of psychological inference in which observation, memory, knowledge, and reasoning are combined together, in order to penetrate the thoughts and feelings of others. The definition by Ickers effectively captures the multi-dimensional nature of empathy and clearly refers its cognitive aspect, namely the comprehension of mental states of the other. In other words, such definition fully comprises the ability of mentalization. The question of the relative overlap between the concepts of empathy and mentalization remains, here, a still-open theoretical point as well as a subject of further study.

As seen above, the cognitive component of empathy consists in the comprehension of a situation from the point of view of another person. This implies taking into account that any other person reacts to one given situation based on his/her beliefs, goals and intentions that can well be different from our own ones. This ability requires skills described by the “Theory of Mind”: when written with capital “T” and “M,” this phrasing indicates processes underpinning specific aspects of the meta-cognitive knowledge. Specifically, we refer here to the ability to recognize ourselves and the others as thinking entities. This means to attribute, to ourselves and to others, specific mental states (e.g., emotions, wishes, beliefs and intentions) and predict future behavior, based on this information.

The origins of the theory of metacognition are linked to work by two primatologists (Premack and Woodruff, 1978) who investigated the ability of chimpanzees to estimate the behavior of a human actor in an experimental paradigm. Some years ago, taking up the ideas of Premack and Woodruff (1978) and Wimmer and Perner (1983) set up an experimental paradigm intended to evaluate the ability of a baby to understand that different people can have a different thought relative to the very same situation. The resolution of this task by children provided the most convincing proof of the capacity to understand the mental state of others.

Different authors during recent years have focused their attention on the ontogeny of the ability to assign a mental state to the others, trying to offer a clear definition of “metacognition of mind.” Despite many authors do agree on the developmental steps that bring to a mature development of a Theory of Mind, different positions still exist when trying to identify the mechanisms and fundamental process subserving these mentalization abilities. The theoretical investigators proposed that a child develops the knowledge on the (own and others’) mind simply on the basis of experience (Botterill, 1996; Gopnik, 1996). This theory supports that ontogeny of the mentalization skills goes along with the mental progress that the child realizes in other fields of knowledge (e.g., communication and language) and depends on the maturation of his/her abilities of representation and symbolization. Studies on linguistic acquisitions in the 1st 2 years of life demonstrate that the onset of interactional communication, the use of communicative gestures and of gaze in joint attention can be considered as precursors of the mentalization ability, allowing to understand the intentional behavior and the mental state of others (Pizzuto and Capobianco, 2008; Capobianco et al., 2017).

In particular, the onset (around 9–12 months) and the use of deictic gestures and of gesture-speech combinations are very important predictors of early development for both Theory of Mind and empathy in children. When a child points by alternating the gaze between an object and the adult, (s)he demonstrates that (s)he already understands an early level of the Theory of Mind: the agentivity of adults. In other words, (s)he knows that the adults’ behaviors can be modified according to his/her purposes. With further development, the child will use the pointing not only to require but also to share an object with the adult (“Declarative Pointing,” see e.g., Capobianco and Cerniglia, 2017; Bartolomeo et al., 2018).

Many studies observe that the gesture-speech combinations encode different information, which represent an important portion of children’s production and play a significant role during the transition from one- to two- and multi-word speech. Recent results on 12 typically developing Italian children, observed monthly between 10 and 25 months, have highlighted that both combination types show a significant developmental pattern during the 2nd year of life, each providing specific contributions in language and communication development. Overall, this entails understanding of mental states and developing a typical interaction and a secure attachment between the mother and child (Capobianco and Cerniglia, 2018a,b; Cimino et al., 2018).

According to “the theory of simulation” (Goldman, 1992; Harris, 1996; Tomasello, 1999), the comprehension of the mind of others is not founded on a complex psychological knowledge of beliefs and wishes of others; rather, it is rather linked to the ability assume others’ standpoint. Accordingly, to deduce the mental state of others would consist in the simulation of the world as experienced by the other. An empirical support to this theory has come from the discovery of mirror neurons: it has been shown that the same neural circuit, involved in the control of action and subjective experience of emotions, is active even when we observe the same actions and emotions while they are accomplished and expressed by others. Starting from this point, Gallese and Goldman (1998) have supposed that the human ability of reading the mental states of others can have evolved rooting on the mirror neurons circuit in primates.

The approach by Baron-Cohen (2011) supports—instead—an instinctual vision for the Theory of Mind and affirms the existence of “specific, genetically-determined and autonomously-functioning mechanisms.” For Baron-Cohen, these “modules” always develop according to a maturation process in which environmental influence is minimized. The development of the comprehension of others’ mind is determined by the activation of a module underpinning the Theory of Mind, termed (toMM, Theory of Mind Module). Such module helps to represent the comparison between one’s own mental state and the others’ mental states, related for instance to the representation of things. Empirical evidences are given by studies on children affected by autism, or other diseases that seem to be characterized by a deficit of those modules.

Finally, we have to mention the integrated socio-constructive approach, supported by authors like Bruner and Feldman (1993). This interactionist and relational approach refuses the classical individualistic perspective in favor of a Theory of Mind that develops in a context of sequences of social interactions. Research by Fonagy and Target (1996) shares such an integrated approach. According to them, mentalization is defined as the ability to understand the interpersonal behavior in terms of shared mental states. This is a fundamental key for the organization of the Self; such affective regulation is acquired in the field of the early relationships of attachment, while all the psychological processes consequent to the ability to mentalize are indicated with the term “reflective function.”

## Neurobiological and Physiological Correlates

### Endocrine and Hormonal Correlates

International research on the neurobiological and physiological bases of empathy agrees on its biological substrate, in particular, supported by the endocrine system (Preston and de Waal, 2002; Yildirim and Derksen, 2012; Gonzalez-Liencres et al., 2013). In this regard, recent studies highlighted some interesting neurobiological elements related to sex (Davis, 1983; Baron-Cohen et al., 2001; Derntl et al., 2010). In fact, several studies showed that women usually get higher results in tasks and social activities in which empathy (both cognitive and emotional) is involved, while men are more likely to suffer from problems characterized by low (or no) empathy, such as psychopathy for instance (Cale and Lilienfeld, 2002).

Such evidence can be better understood considering the presence of higher levels of testosterone in men compared to women; this difference could partly explain the variability in empathy related to sex. In fact, affective empathy results to be influenced by the activity of the amygdala, whose functioning, in turn, depends on testosterone levels (Terburg et al., 2009). The association between testosterone levels and empathy deficits seems to play an important role in autistic disorder which, according to one theory, is even attributable to fetal testosterone (Lutchmaya et al., 2002; Baron-Cohen et al., 2005; Chapman et al., 2006).

For a better understanding of the neurobiological mechanisms of empathy, it is important to highlight that, in addition to testosterone, other elements are found to play a relevant role: these include, for instance, the release of glucocorticoid under stressful conditions *via* the hypothalamus-pituitary-adrenal (HPA) axis (Tennes and Kreye, 1985; Adam and Gunnar, 2001; Stallings et al., 2001; Nakayama et al., 2007; Booth et al., 2008; Barraza and Zak, 2009; Buchanan et al., 2012).

Oxytocin (OXT) is a nonapeptide with a long evolutionary history and a well-established role in animal and human social cognition. Brain regions clearly associated with social cognition include amygdala, insula, medial prefrontal cortex (mPFC), anterior cingulate cortex (ACC), temporoparietal junction, superior temporal sulcus (STS) and inferior parietal lobule (IPL). Research (Mariscal et al., 2019) suggests that children with autism spectrum disorder (ASD) may suffer reduced empathy, as measured by impaired yawn contagion, compared to typically developing (TD) children. Blood concentrations of the “social” neuro-peptide OXT positively predicted contagious yawning. These findings suggest that OXT identifies a biologically defined subset of children with ASD, who exhibit reduced empathy as measured by impaired contagious yawn.

Another study (Luo et al., 2019) tested the hypothesis that empathic neural responses to others’ pain are more flexible in G/G than A/A carriers (rs53576) of the oxytocin receptor gene (OXTR). They found that G/G carriers showed greater neural responses, over the frontal cortical region, to painful vs. neutral face expressions. In contrast, A/A carriers showed significant empathic responses only to faces with shared racial and group identity. These findings provide electrophysiological evidence for interactions between OXTR and intergroup relationships about empathy for others’ suffering. Another study (Acevedo et al., 2019) used magnetic resonance imaging (MRI) to visualize brain areas responsive to happy or sad faces: evidence shows that OXT increases the ability to recognize emotional expressions in faces. Moreover, some studies found a specific pharmacological effect of OXT administration on the recognition of happy faces, while others found a specific effect for the recognition of fear (Domes et al., 2007; Schulze et al., 2011). Intranasal application of OXT has been shown to increase emotional, but not cognitive empathy, potentially by an amygdala-dependent mechanism (Luminet et al., 2011). The “Reading the Mind in the Eyes Test” (RMET) requires the ability to detect emotional states from pictures that are exclusively depicting the eye region of individuals (Baron-Cohen et al., 2001): this test covers socially cognitive functions, namely mentalizing. In this specific paradigm, administration of 24 IU of OXT improves performance (Fischer-Shofty et al., 2010). This effect is specifically observed in individuals with a baseline of higher alexithymia or reduced empathy, and therefore points once again to the role of OXT in empathic abilities.

### The Neurobiological Bases of Empathic Ability

Studies in the field of biological basis of empathy have identified a series of neuronal systems that support the ability of subjects to enter into relationships, both between adult subjects (romantic relationship) and between parents and children (parenting relationship). In particular, embodied simulation and mentalization refer to the ability of subjects to observe, and care for, the other. In support to the relevance of these evidence, recent studies have shown that sensitive parenting influences the psychological development and well-being of children during development (Ammaniti et al., 2004; Tambelli et al., 2015).

Many studies in the literature show the complementarity of mother-children neuro-biological systems (Lenzi et al., 2013). For example, studies have shown that parents are willing to take care of their children in response to the global characteristics of her/his face, which acts as a particularly attractive configuration-gestalt (Darwin, 1872; Glocker et al., 2009; Kalikow, 1983; Stern, 1977). This seems to be linked to the tendency in adults to protect a child and help her/him to survive, transmitted during the course of evolution.

Several areas of the brain have been recognized as a biological substrate of empathy (Gallese and Goldman, 1998; Carr et al., 2003; Lenzi et al., 2013). Among them, the parieto-frontal cortical circuit assumes a crucial role: its activation, when a subject observes the action of another subject, demonstrates the biological basis of the ability to reflect the behavior of others and the related emotional content (see Rizzolatti and Craighero, 2004; Rizzolatti and Sinigaglia, 2010).

As anticipated above, the neuroscientific research on empathy is highly influenced by discoveries of a group led by Giacomo Rizzolatti who, in the middle of the 90s, described a population of neurons in the pre-motor area F5 of the macaques’ brain, afterwards called mirror neurons (Gallese et al., 1996; Rizzolatti et al., 1996). These were activated when the ape performed an action with the hand but also when the ape observed the same action performed by another one. This discovery showed the presence, in the primates’ brain, of a transduction mechanism: when apes observe the others’ actions, mirror neurons have been demonstrated to be elicited also in terms of anticipating the corresponding motor control. As such, this mirror system transforms what they see in immediate knowledge on what to do.

Transcranial magnetic stimulation (TMS) data in humans have been used to infer the role of mirror neurons. Fadiga et al. (2005) suggest that, when a subject observes another individual while producing some action, (s)he is somewhat stimulated to activate the same muscles. Gallese and Goldman (1998) suggested that the mirror neurons’ system can constitute the neurobiological basis which underlies our ability to understand the others and to simulate automatically their possible actions and intentions. There is a wide neuroscientific literature, nowadays, supporting the idea that simulative processes could constitute a basis of our social brain and our ability to empathize with other people (Preston and de Waal, 2002; Gallese et al., 2004). The mechanism that enables the sharing of a mental state with other people has been extended to the ability of sharing feelings and sensations (Gallese et al., 2004). To support this hypothesis, neuroimaging studies have investigated the brain activity associated to different empathic responses in the field of contact, smell and pain. These studies will be briefly reviewed here.

One of the first affective state that (as shown by neuroscientists) implies a form of empathic comprehension is the *disgust*. The neurons of the anterior insula (AI), a portion of brain cortex are activated when the subject shows disgust to olfactory and gustatory stimuli. Studies (Wicker et al., 2003) have shown that the AI is activated not only in presence of these stimuli but also when we are seeing other subjects that show facial expressions of disgust. This mirror role of the insula has been confirmed with neuroimaging and clinical studies: it has been observed that a lesion of the insula not only causes an impairment in the abilities of feeling disgust but also impedes the ability to recognize the facial expression of disgust by others (Gallese, 2001). According to Rizzolatti and Sinigaglia, this result suggests that the real comprehension of disgust by others is not based on an inferential cognitive process but on a mirror empathic process. The studies on touch have shown similar evidence for the activation of the somato-sensory cortex, involved in the elaboration and in the perception of touching. This happens both when subjects are touched by others but also when they observe other people that are touched (Keysers et al., 2004).

Very interesting results were obtained, by researchers, on the capacity of empathy to feel others’ pain. It has been posited that a complex brain circuit called “matrix of pain” is involved in the experience of pain. The matrix of pain includes the primary and secondary somato-sensory cortex, the cerebellum, motor and premotor cortices. The affective knot of the matrix includes the ACC and the insular cortex (IC): these are phylogenetically ancient regions, classically considered components of the limbic system and of the visceral brain (Papez, 1937; MacLean, 1949). Singer et al. (2004) measured the activation of such neural structure in 16 women to which the painful stimuli (slight electric shocks on the hand) were given in a first moment; in a second moment, they watched the administration of similar painful stimuli to their fellow subjects. Some brain areas, such as the front of the insula, the ACC, the cerebellum and the brainstem, activated both during one’s own experience of pain and in the perception of the pain in others; instead, other areas as the primary and secondary somato-sensory cortices, activated just by own pain. The authors concluded that empathic experience of others’ pain consists in sharing their subjective experience of pain, without sharing the sensorial elements.

A recent neuro-imaging study (Novembre et al., 2015) shows that the social pain involved the posterior insular cortex, a brain area traditionally associated with the elaboration of physic pain: the experience of social pain would occur both when we feel it in first person but also when we experience it in an empathic way. Another indication of a neural activity linked to pain, in the absence of physical painful stimulation, came from a neuro-imaging study (Osaka et al., 2004). In an experimental paradigm, the recruited subjects listened to onomatopoeic words in Japanese that evoked pain; subsequent to this an increased magnetic-resonance signal within the ACC was observed, which suggests a real affective representation of that pain (Singer and Fehr, 2005).

Importantly, research on the above neurobiological correlates of human empathy has significantly contributed to the development of a bio-behavioral synchrony model (Feldman et al., 2012) positing that parental (mature) psycho-physiological system externally regulate the infants’ immature system by coordinating and modulating biological and behavioral processes during social contact. It has been shown that multiple neuro-physiological systems (e.g., hearth rate coupling, brain-to-brain synchrony in neural oscillations, endocrine system) are anchored in behavioral synchrony and enhance the moments of attunement and concordance between parents and children during verbal and non-verbal interactions (Levy and Wagner, 2011; Cerniglia et al., 2018).

In line with this perspective, evolutionary theories, psychoanalytic theories of affect, developmental theories, social learning theories and attachment theory, all agree that parental empathy is the key component of sensitive parenting and, therefore, of children’s social adaptation (Abraham et al., 2018). Several crucial capacities in children (e.g., affect regulation, socialization, cognitive functioning, symbolic competence) are predicted and supported by their parents’ empathic ability (Feldman, 2007), which enables offspring to feel secure, show compassion towards others, and recognize their own thoughts and feeling (Fonagy et al., 2007). By modulating physiological response to stressing signals and experiences (e.g., modulating the release of cortisol in children), sensitive and emphatic parenting supports emotion regulation processes in children (Knafo and Plomin, 2006; Muratori et al., 2011), and in general is associated with a positive development of both cognitive and affective empathy in children (Miklikowska et al., 2011; Soenens et al., 2016). In fact, a positive caregiving can facilitate the emotional growth of children and adolescents: they will become able to consider the point of view of their peers, to care for their experiences, and to engage in prosocial behaviors (Hastings et al., 2007). Many authors have highlighted the link between difficulties in empathy and difficulties in the relationship between parents and offspring, with particular reference to the children’s problems in maintaining a visual contact and joint attention with their parents (Dadds et al., 2014; Billeci et al., 2019). Following these considerations, several clinical psychologists and researchers have linked children’s empathy deficits to negative experiences with caregivers, including forms of maltreatment. Empirical data about these aspects are not conclusive: some children and adults who have experienced maltreatment show normal or even high emotional capacities, whereas others display emotional and cognitive deficits (Dadds et al., 2012). The fact that data collected in this field of studies are not homogeneous highlights the particular complexity of the construct of empathy that can be influenced by genetic and environmental variables (Dadds et al., 2009).

Most importantly, human capacity of empathizing is crucial for all social interactions, not only those between children and their parents (Eisenberg and Miller, 1987). Bio-behavioral synchrony, the coordination of biological and behavioral processes between attachment partners during social contact, is a critical component of all human attachments (parental, romantic, friendship, and fellow–human interactions), although synchronous interactions experienced during early sensitive periods are expressed in later attachments throughout life. Dopamine striatal neurons are suggested to support this synchrony because their temporal sensitivity may allow humans differentiating reward that stems from self and/or partner, anticipating predictable social interactions, and drawing reward from the matching of self and partner’s actions (Báez-Mendoza and Schultz, 2013). Recent studies tested the neural basis of humans’ multiple attachments by measuring brain responses to auditory, visual, or multimodal stimuli of the attachment target (e.g., infants to their parents, romantic partners, or friends). It has been suggested that the VTA (ventral tegmental area)/ventral striatum ignites the brain’s experience of attachment and imbues it with reward and vigor, and that human attachments integrate subcortical with multiple cortical reward- and socio-cognitive-related networks (Lieberwirth and Wang, 2016). On one side, the fronto-striatal connections of the reward circuit, particularly the ventromedial prefrontal cortex (vmPFC), the orbitofrontal cortex (OFC), and the ACC seem to support human capacity of establishing and maintaining intimate relationships by sustaining bio-physiological and psychological synchronicity and empathy (Abraham et al., 2016). On the other side, the embodied simulation/empathy network, which includes the insula, the ACC, the inferior frontal gyrus (IFG), the IPL, and the supplementary motor area (SMA), is suggested to be critical for grounding a “shared world” in the brain underpinning the human capacity to build and maintain attachments (Frith and Frith, 2006). Besides, human bonds also rely on higher-order (mentalizing) cognitive processes, supported by fronto-temporal–parietal structures, specifically the STS, the posterior cingulated cortex, the temporo-parietal junction (TPJ), the temporal pole, and the mPFC.

Apart from these studies, a particular type of cell has been also observed, called the von Economon’s neurons that are present only in the ACC and in the AI of humans. Allman et al. (2011) believes that this neuron could constitute a neural basis for mechanisms underpinning knowledge about the one’s own and others’ roles and interactions within complex social settings. Thus, the same structures that play a decisive role in the representation of own affective states (like AI, ACC) seem to have a decisive role in the comprehension of the affective state by others.

During the last 10 years, many studies with functional neuro-imaging on healthy adults have focused on investigation of the ability to mentalize (Ruby and Decety, 2003; Singer, 2006). The results have shown the existence of a neural network underpinning the ability to understand and predict the behavior of others, possibly through the assignment to them of mental states. This network includes three brain areas that are greatly involved: TPj and temporal poles (TP), upper posterior temporal sulcus (pSTS) and, above all, the mPFC (Farrow et al., 2001). Given the heterogeneity of methods, and of the demographic characteristics of participants recruited in these studies, it is remarkable that the results converge on the same areas of the brain. According to the vision currently shared in literature, the three brain area supporting the Theory of Mind play different functions: the STS could be responsible for detecting and recognizing the biological movement of others, as the direction of gaze, the reading of lips and the movement of body, hands and lips; the TP, associated to memory processes, give the semantic context in which stimuli are elaborated; finally, the mPFC is implicated in the subsequent analysis of such stimuli and produce explicit representations of one’s own and others’ mental states (see Fletcher et al., 1995; Gallagher et al., 2000; Cuff et al., 2016).

## Emotions vs. Cognition (or Both)?

### The Subcomponents Within Theory-of-Mind

Although the topics dealt with by the theory about metacognition of mind have gained attention in cognitive neuroscience during the last 20 years, only recently the experimental research started to examine subcomponents of such complex activity. Therefore, it is important to be aware that the different roles of its neural correlates are currently investigated, at least for what concerns the prefrontal cortex (PFC). Shamay-Tsoory et al. (2009, 2010) proposed a model for the Theory of Mind in which two separated systems were involved: one elaborates the inferences on beliefs and intentions of others (Cognitive Theory of Mind) and the other realizes an inference of emotions and feeling by other people (Affective Theory of Mind).

A series of studies on lesions (Shamay-Tsoory and Aharon-Peretz, 2007) have shown that patients with lesions to the vmPFC have a deficit in tasks that involve an affective component, as the comprehension of the affective state hidden behind an ironic comment in a non-affective context. These observations support the hypothesis that vmPFC could be necessary for the affective empathy but not for the cognitive one. This area has many connections with regions involved in the affective elaboration (amygdala, AI and TP). Thus, it may integrate cognitive and affective information. A support to this hypothesis comes from the studies on patients with psychosis (see Shamay-Tsoory et al., 2010): a deficit in empathy was shown in correspondence to dysfunctions of the amygdala and of the OFC.

One neuroimaging study explored the overlapping neural correlates of cognitive and affective Theory of Mind, hypothesized by Shamay-Tsoory. The study (see Sebastian et al., 2011) has inquired into the relationship between the affective and cognitive subcomponents with illustrations that predicted separate intentional conditions for the cognitive vs. affective mind, plus a condition to control for physical causality. Results have shown that both cognitive and affective capability of inferring others’ mental states activate brain areas, such as the posterior STS, the TPj, precuneus, and temporal lobes, thus confirming the model (Shamay-Tsoory et al., 2010).

An important contribution about PFC’s different roles in Theory of Mind was given by a recent study (Xi et al., 2011), who used a complex task, the Faux Pass Test (Baron-Cohen et al., 1999), that comprehends both affective and cognitive subcomponents. This test puts participants in the position of reading a story in which the statement-making character does not realize that he should not have expressed such affirmation and that listeners could have felt offended. Subsequently, participants are given six questions, for evaluating their ability to recognize the Faux Pass as well as their empathic capabilities and mental representation.

Results of the study evidenced that patients having vmPFC lesions did not recognize the gaffe correctly (affective component of the test). Conversely, patients with dorsal/lateral PFC lesions recognized the gaffe correctly but did not manage to infer the other’s mental state (cognitive component of the test). The affective mind also involved the vmPFC, providing a validation of the hypothesis that affective Theory of Mind requires additional elaboration compared to cognitive Theory of Mind: vmPFC’s activation reflects the integration of cognitive and affective information.

### Differences and Overlaps Between Empathy and Theory-of-Mind

In neuroscientific and cognitive literature, there is often a clear difference between the Theory of Mind and empathy. On one side, empathy concerns our ability to share affective states with others; on the other hand, Theory of Mind represents our ability to interpret their mental state, their intentions and beliefs (Blair et al., 1996). The distinction between the two concepts is supported by the evidence of social impairments in people with psychosis: indeed, people with a diagnosis of schizophrenia or under drug-induced psychosis show inability to empathize but they do not show any deficit in mentalizing. The dichotomy between empathy and Theory of Mind distinguishes between intentions and beliefs, on one side, and emotions on another side. Rational vs. affective domains of life are indeed the two poles if such a dichotomy.

According to this model (see also above, “Endocrine and Hormonal Correlates” section), the affective empathy, after a sensory perception of emotions in others, occurs through the same neural mechanisms that would be activated and are responsible for the emotional experience in first person; thus, empathy would be based on mirroring processes like emotional contagion, shared pain, recognition of emotions. The cognitive facet of empathy, instead, consists of the ability to “infer”—using cognitive skills—the emotional state of others: according to the model by Shamay–Tsoory, mentalization involves both cognitive and affective processes, and specifically, affective empathy refers to the possibility of inferring others’ emotions.

With reference to the studies of Shamay–Tsoory, empathy is composed by two components, affective and cognitive, while the Theory of Mind is referred to our ability to get inferences on others’ beliefs but also emotions. By functional neuro-imaging Schnell et al. (2011) investigated neural correlates of “cognitive” empathy as defined by Preston and de Waal (2002). These authors highlighted how this mechanism of emotional representation is still relatively unexplored and connects to the (cognitive) domain of the mentalization system.

A differential study was realized with illustrated stories of faux beliefs in first and third person, in which all direct signals depicting affective states (like the face’s expression of all characters of the story) have been deleted. Results confirmed the hypothesis that cognitive empathy involves, at the same time, the neo-cortical network of mentalization and the limbic areas involved in the experience of emotion in first person. This implies that cognitive empathy entails a “simulated” generation of an affective state through activation of the limbic system, in the absence of an external (direct) emotional signal.

The general involvement of simulation in mentalization is moreover supported by studies of Lombardo et al. (2009). Limbic simulation can explain the mirroring of others, i.e., elaboration of affective empathy; mentalization, in its affective and cognitive subcomponents, could support cognitive empathy. A reduction of empathy, according to this model, could be caused by a deficit of either mirroring (limbic simulation) or affective inference (mentalization), that are mediated by different neural systems.

From a neurobiological point of view, when humans are required to solve cognitive tasks that imply empathy or Theory of Mind, different neural structures are activated. Shamay-Tsoory’s theory distinguishes two different capacities for the comprehension of others. Such two forms of comprehension are interconnected, from both a psychological and a neural viewpoint, since neo-cortical or limbic brain areas often interact with each other.

Völlm et al. (2006) studied this overlap (in neural correlates of empathy and Theory of Mind) and found that the comprehension of others’ emotional states required the involvement of other interconnected areas, in particular, the amygdala and the orbital–PFC.

### The Full Interaction Between Empathizing and Mentalizing

According to the model proposed by Shamay–Tsoory, our human interpersonal relationships in normal social circumstances would activate, independently, both a mirroring of the emotional response (emotional empathy) and a cognitive evaluation of the others’ affective mental state (cognitive empathy; Shamay-Tsoory et al., 2009). Several evidence seem to confirm the existence of such different pathways, acting in parallel and relatively autonomous, allowing the full experience of empathy (Eres et al., 2015; Valk et al., 2016).

In line with a “theorethical/deductive” approach, people use mind deductions to attribute intentional states to others; that is, they adopt a fully theoretical-reasoning approach for the comprehension of others’ mental state, relying onto a mechanism of mentalization. According to the “Theory of Simulation,” instead, we attribute mental states through a process of intuitive mirroring. This perspective emphasizes the use of shared representations: thanks to the innate faculties of observation-execution, the emotion felt by others is shared empathically. The system of mirror neurons would be particularly suited for providing a simple mechanism of motor empathy, like for imitations and emotional contagion. In an independent way, the deductive processes would be activated to infer the perspective and the mental state of others: it would require the ability of distinction between self-else and recruit the autobiographic memory. Therefore, a final cognitive empathic response would be generated, involving—in parallel—the cognitive mind (mPFC, STS, TP) and the affective mind (vmPFC, OFC). On the contrary, an emotional (“instinctive”) empathic response is simply guided by simulation and involves the same regions that mediate the first-person emotional experience (amygdala, insula).

Even if the emotional and cognitive components of empathy work autonomously, every empathic response would activate the same components, as a function of different variables including social context, the level of stress (Jackson et al., 2005) and the perceived similarity between the observing subject and the observed person (Mitchell et al., 2005). Therefore, although both the affective and cognitive components of empathy can work independently, in any natural social situation every empathic response involves both components (Zaki and Ochsner, 2012). We can assume that the balanced activation of such networks is fundamental for an adaptive social behavior. As the system of mirror neurons seems to be of a lower level, one could think that its activation could represent a prerequisite for the activation of the more complex mentalization system.

Van Overwalle and Baetens (2009) have suggested that the mirror neurons and the mentalization systems are rarely active at the same time: it is possible that the mirror-neuron system provides a fast and intuitive input to the mentalization system. But social interactions themselves represent the privileged condition to explore the potential connection between the two systems. Social interactions are in fact characterized by a continuous exchange of roles between actors; this exchange, of course, not only the comprehension of the other’s action but also the capability to infer other’s intentions. To the purpose of studying this dynamics, which seems to require a connection between mirror neurons and mentalization systems, Sperduti et al. (2014) realized a study in which two subjects underwent functional magnetic resonance while involved in a reciprocal game of imitation of each other’s hands. The two participants could observe each other’s hand movements and they were free to choose whether to imitate each other’s movements or to freely produce some gestures at any moment. To produce a harmonic exchange of roles, each subject needed to anticipate the other’s intentions. Results have evidenced a relationship between the functioning of mirror neurons and mentalization systems. During active (imitating) and passive (to be imitated) “imitation” conditions, when compared to a passive observation condition, functional neuroimaging has shown a major activation in the IFG concurrently with ventral and dorso-medial prefrontal cortices and with the ACC.

In sum, the mirror-neuron system is involved into the preparation of one’s own actions and into the simulation of the other’s actions, while the mentalization system is likely involved in higher-level cognitive processes, that allow the anticipation of the other’s intentions, a fundamental step in the co-regulation of reciprocal action. A recent review (Catmur, 2015) has expanded on the role of mirror neurons and mentalization systems: mirror neurons would be majorly activated in the perception of action, a process that could be necessary but not sufficient for the full comprehension of the intention. Another recent research on comprehension of others’ intentions (Isoda, 2016) supports the idea that mirror-neuron system is activated by others’ visible actions, and predicts physical consequences in terms of scopes, while mentalization system would be mainly involved into the forecasting of others’ intentions. Interestingly, impending actions would be detected regardless whether others’ current actions can be observed directly or not. Isoda suggests the hypothesis that the two systems strictly collaborate to judge the meaning of the others’ actions.

A speculative conceptual model, that allows to comprise the most critical aspects of social cognition, includes formulating statements about others’ intentions and comprehending the meaning of their actions. Tidoni and Candidi (2016) evidence the difficulties of demonstrating that mirror neurons and mentalization areas contribute separately to the perception of actions and to the comprehension of others’ intentions. Another recent and interesting research (Kanske et al., 2016) has further looked into the relationship between emotion sharing and the comprehension of others’ mental state during social interactions. People with higher levels of empathy do not necessarily possess a greater mentalization capability. In highly emotional situations, though, empathic sharing could inhibit the activity that is bound to mentalization and therefore degrade the mentalization performance.

Data would, therefore, suggest the existence of two distinct social systems interacting with each other rather than a unitary construct of social comprehension. The AI takes on a more instinctive and visceral reaction about what’s happening to others, with a role in modulating current processes within TPJ, such as the undertaking of the others’ point of view or reasoning about the others’ mental state. In a very recent study with functional magnetic resonance, Buades-Rotger et al. (2017) have investigated the role of brain areas associated with aggressive or avoidant responses to interpersonal provocation. The participants are put in situations where there is choice of facing or avoiding an opponent with a high (HP) or low (LP) level of provocation. The brain areas containing mirror neurons have shown a major activation when the participant decided to face the opponent, while mentalization circuits were more involved in the avoidance decision. Moreover, avoidance of HP opponents was associated to a major activation of amygdala and to a slightly lower activation of mentalization regions (IFG, TPJ), compared to avoidance of an LP opponent. Such results would suggest that avoidant responses are associated to a minor assumption of perspective and could represent the outcome of a major anticipation of threatens. Data also suggests that the two systems play a convergent role in decisions which regard response to provocation. However, there is a necessity of causal evidence from new social-interaction studies, proposing different conditions under which each one of them may activate the other one.

### Empathy and Psychopathology

Research shows that many psychopathological forms are characterized by difficulties in empathy. In particular, Smith (2006) proposed a distinction into four empathy disorders. These disorders are: *cognitive empathy deficit disorder* (CEDD), characterized by low cognitive empathy and high affective empathy; *emotional empathy deficit disorder* (EEDD), characterized by low affective empathy and high cognitive empathy; *general empathy deficit disorder* (GEDD), characterized by low abilities in both cognitive and affective empathy; *general empathy surfeit disorder* (GESD), characterized by a high level of both cognitive and affective empathy.

The CEDD type of empathy disorder is associated with ASD and Asperger’s syndrome, both of which are characterized by difficulties in understanding the mental and emotional state of a subject (Capps et al., 1993; Smith, 2009). But several clinicians and researchers do not agree on the presence of a unique deficit of empathy at an emotional level in autistic subjects. Also, many authors do not agree with the understanding of autism based on the Smith model, which seems to be too rigid and limiting if considering a greatest variability of phenotypic manifestations within the autistic spectrum (Shamay-Tsoory et al., 2002; Minio-Paluello et al., 2009). Moreover, in this regard, several studies showed the presence—in autistic subjects—of difficulties in empathy even at the lowest levels of cognitive processing, with errors in the simple identification and recognition of facial expressions (McIntosh et al., 2006; Clark et al., 2008).

The CEDD category of empathic disorder has been associated not only with autism but also with other disorders, including fronto-temporal lobar degeneration (FTLD), bipolar disorder and borderline personality disorder (BPD; Harari et al., 2010; Cusi et al., 2011; Mier et al., 2013). In addition, the other typologies of Smith’s classification have also been associated with various disorders. More specifically, psychopathy and antisocial personality disorder (ASPD) seem to correspond to EEDD, because the subjects understand the emotions of others but are not able to socialize with them (see Dolan and Fullam, 2004; Decety and Ickes, 2009; Schwenck et al., 2011), whereas schizophrenia seems to be categorized as GEED, as the subjects show difficulties in understanding the mental and emotional state of others from both an emotional and cognitive level (Bora et al., 2008; Haker and Rössler, 2009).

Moreover, several studies in patients with brain lesions have confirmed the usefulness of distinguishing between various empathy disorders. For example, cognitive-empathy deficits were found in patients with lesions at the level of the vmPFC, while emotional-empathy deficits were found in patients with lesions in the lower frontal gyrus cortex (IFG; Shamay-Tsoory et al., 2009). Still, the well-known area 44 of Broca has been identified (by neuroscientists) as neurobiological correlate of affective empathy and of the mirror neuron system, which is particularly important in sharing mental states between subjects (Rizzolatti, 2005; Shamay-Tsoory et al., 2009). On the other hand, though, the association between difficulties in empathy and pathologies cannot always be univocal, as there are also a number of psycho-pathological conditions that cannot be traced back to the specific type of empathy deficit nor to the categories of the Smith’s model. This is the case, for example, of attention deficit and hyperactivity disorder (ADHD) and related worsening like e.g., conduct disorder (Braaten and Rosén, 2000).

Taken as a whole, the elements of evidence on the neurobiological foundations of empathy analyzed so far recall the need for future investigation on the sub-types of emotional and cognitive empathy, and on their characterization within different psychopathologies. This effort can turn out to be particularly useful, e.g., to organize prevention and intervention programs effectively based on the characteristics of cognitive and relational functioning of the patients.

## The Contribution by Animal Modeling

Today, the utility of animal models is recognized in neuroscience. Laboratory rodents can very easily learn how to produce a behavioral response in order to produce a given desired effect, and many “operant” tasks have been developed. Interestingly, however, these subjects are often tested individually; alternatively, they can be tested while living in group, like e.g., within IntelliCages: however, the measured behavior usually still produces effect for the subject itself. Rodents are rarely confronted with situations in which the effect of own actions is tapping onto another subject (and vice versa). When such protocols are proposed, there are the concepts of metacognition popping up. To what extent indeed does the acting subject demonstrate, by observable actions, to select those actions based on own “empathy”?

As far as preclinical modeling is concerned, with the term “empathy” we mean the ability to identify the mood of other conspecifics. This is based on:

1. understanding their emotional signals, which allows to foresee in advance their intentions;
2. sharing their feelings (e.g., mirroring expressions of disgust or pain);
3. assuming their subjective perspective, i.e., role-taking.

We hereby propose that evidence of modulation in deeds by one subject based on the emotional state of the other subject in a dyad can be used to infer an empathic response. As a matter of fact, among definitions of empathy described above, it was assumed that the behavior of one subject would be influenced after getting the (correct) insight about the mood of the other subject. In other words, observable behavior may well adapt to the necessities of the other if such needs are correctly inferred. Therefore, in those paradigms whereby one subject is satisfying the needs of another conspecific, we could well modulate prosocial responses by this one subject by simply changing the conspecific or its emotional status.

Prosociality, or “prosocial behavior,” is a construct that includes all those actions which can produce, maintain or increase the benefits (or well-being) of other individuals, often bringing lack of benefits or even disadvantages for those who perform them (Roche Olivar, 2002). For this reason, it is strongly linked to concepts of altruism and generosity that—indeed—disregard possible benefits. In naturalistic conditions as well as in the wildlife, prosocial behaviors that bring benefits to individuals related to each other are often observed: this happens because animals tend to favor the survival of other individuals who have a similar genetic background and behave so that to increase their fitness accordingly. Prosociality settings in most laboratory protocols are often measuring helpful donations, namely not related to the possibility of obtaining, with these disinterested behaviors, secondary benefits like a courtesy back in the next future.

It has long been recognized that prosocial behavior is widespread in non-human species. The first animals in which prosocial behavior was observed were bats. These nocturnal rodents normally go on excursions to get a blood meal: if an individual fails to eat, it is often fed by a conspecific who regurgitates part of her/his own meal, in favor of the fasting and hungry subject. In addition, individuals who have shown prosocial behavior in the past are fed more frequently than those who have never shown, or not yet, a prosocial behavior.

To understand how rats can be studied for prosocial choice, Márquez et al. (2015) developed a two-choice task in which prosocial behavior did not yield a benefit nor a cost to the focal rat. They used a double T-maze in which a focal rat controlled access to the food-baited arms both of its own maze and the recipient rat’s one. In this task, the focal rat could freely choose between one side of the maze, which yielded food only to itself (selfish choice), or the opposite side, which yielded food both to itself and to the recipient rat (prosocial choice). Rats showed a high proportion of the latter choices. By manipulating the recipient’s ability to display a preference for the baited arm, the authors found that the display of food-seeking behavior by the recipient was driving prosocial choices of the focal rat. Thus, rats provide to others access to food, in the absence of direct self-benefit.

Once established this, the next step is to ascertain whether rats can judge the entity of such donations in comparison to own reward. “Inequity aversion” is defined as the resistance to inequitable outcomes, which occurs in humans when subjects hate inequality. In the case of lab animals, this concept implies that a focal subject is not willing to provide another subject with a reward being much greater than own one. In some instances, inequity aversion can be disadvantageous: in order to prevent another animal from receiving a superior quantity or quality of a given reward, focal animals may be willing to skip an own gain.

The apparatus exploited by Douglas et al. (2018) was composed of two identical operant boxes, placed side-by-side on a table: they had food cups at the center of the wall and a food-pellet dispenser connected to a lever, which released 45 mg sucrose pellets in the recessed food cup. The sample was composed of adult, male rats and a specific goal of the article was to examine how a downward shift in reward value, with food acquisition occurring in a social context, would impact individual operant responding (inequity aversion).

Animals were exposed to different conditions, including a “together” (TOG) condition: two animals placed in the two chambers simultaneously, next to each other, could observe the other rat and hear the different tones. Following a lever press, during a trial, the subsequent outcome was a high-value reward. After the control rat completed one trial, a trial then began for the experimental rat: this schedule continued back and forth until each rat completed twenty trials. Then, a socially-negative contrast was tested in that the session began with a string of five high-magnitude rewards, followed by 15 low-magnitude rewards, for one rat. The neighbor animal continued to receive the high reward outcome. As such, the first (experimental) rat was expected to “suffer and react” because of such inequality. To summarize the overall results, performance in this TOG condition appeared slower than the “alone” control one, when the experimental rat completed the session with no subject nor program running in the adjacent cage.

Although this approach does not address prosocial behavior as no donation is involved, it is quite evident that the experimental rat, while observing the neighbor rat to perform lever pressing and eating the food pellets, and while considering magnitudes of this reward compared to own one, shall be supposed to express a metacognitive skill. This rat is somewhat detecting the intentional actions performed by the other rat to earn food and somewhat considering the level of reward and/or of satiety states, gained by the other compared to own one; as such, elements of mentalization and/or of empathy are somewhat involved.

As far as empathy-driven responses are concerned, some experiments were recently run to observe whether rats were motivated to help a conspecific that showed signs of stress. In particular, in the study by Ben-Ami Bartal et al. (2011), a free rat was placed in an arena where a companion was trapped in a container that could only be opened from the outside. After a few sessions, the free rat learned to intentionally and quickly open the container to help a cage mate. In support of the hypothesis that the intention of the rat was indeed to release the trapped mate, it was observed that rats did not open the container when it was empty or with an inanimate object inside. In addition, rats released their cage mates even when social contact was prevented. Therefore, rats behave prosocially in response to the suffering of a conspecific: as a matter of fact, the subject shall *first* detect the suffering of the other and *then* act to help it. These studies are hence providing important evidence of the biological roots of prosociality as an empathically-motivated behavior.

Silberberg et al. (2014) devised another similar experiment, in which a rat could release a conspecific trapped in a tube by allowing it access to a space located quite away. This protocol was chosen to exclude that the free rat could release the conspecific just to seek for social contact. It rather emerged that free rats are empathically motivated to reduce the discomfort of the trapped rat also when they become very distant after the latter issuing the tube.

Sato et al. (2015) evaluated whether rats do help stressed conspecifics who are wet with water. In a first experiment, rats quickly learned to open the door, allowing the wet trapped rat to enter the safe area escaping from the water area. In addition, it was seen that rats that had previously been wet learned faster to open the door to the trapped partner than rats that had never been wet. This, probably, may happen because the former were able to recognize the unpleasant situation of the latter conspecific. In a second experiment, rats had to choose between opening the door to help the stressed partner and opening a different door to obtain a food reward. In most cases, rats would choose to help their partner before they got their food reward. These results suggest that rats behave prosocially; their intention to help may be motivated by states of emotional empathy towards stressed companions.

Also, in the rat there are evidence of reciprocity in prosocial behavior. In pioneer study, Rutte and Taborsky (2007) showed that the cooperative behavior of rat females is influenced by having previously received help, regardless of the identity of the partner. Rats were trained to pull a stick so to provide food to the partner. Test subjects threw it more often for an unknown partner when they had been helped than in situations when they had not previously received help. This phenomenon, called generalized reciprocity, does not require specific knowledge of the partner and may promote the evolution of cooperation between unrelated and unknown individuals. Subsequently, Dolivo and Taborsky (2015) have shown that rats are able to distinguish between different collaborators based on the quality of their help. Their data confirm that rat’s propensity to reciprocate aid is proportional to the quality of aid previously received by the same partner. To sum up, rats are more likely to help a conspecific they do not know but who previously helped them.

The aim was then to understand factors on which rats rely, in order to decide when they should return the aid received (Dolivo and Taborsky, 2015). In fact, even when visual information was missing, rats provided more food to partners who had previously helped them to obtain food than to those who had not. In a similar way, Hernandez-Lallement et al. (2015) trained pairs of rats—an actor and a partner—to decide between two alternatives that differed only in the reward given also to the partner. With the “own reward” choice, the reward was accessible only to the actor, while the “both reward” choice also produced a reward for the partner or for an inanimate toy, placed in an adjacent compartment. These authors found that the actors chose the “both reward” option significantly more than the random level and more often in the condition with the real partner than in the condition with the toy. This study shows how rats are influenced by the presence of a conspecific in implementing a behavior that produces also a benefit for the other.

Last but not least, a study by Li and Wood (2017) determined if unrelated male laboratory rats would respond *on behalf* of a partner in an iterated sequential game. Trial-by-trial analysis of “donor” and “responder” responses provides evidence that rats are not following a strategy of generalized reciprocity, but are sensitive to responses of their partner in previous trials. The study revealed that, when rats were paired with their cagemate, the responses were consistent with reciprocal altruism. By contrast, when paired with another unrelated partner (a stranger), and even when paired with a maximally-generous partner (good stooge), response rates decreased, since test rats failed to adjust their donor responses to obtain more pellets. These findings, obtained with a “repeated donation” game, seem to align with previous studies of sequential cooperative behavior in primates.

These results are consistent with direct reciprocity, whereby participants work on behalf of a partner who has worked for them previously. This suggests however that rats, when playing an iterative sequential game, are more likely to respond on behalf of a cagemate. Nevertheless, rats show direct reciprocity for a partner who previously provided a food reward and respond more rapidly for a partner who provided a preferred reward. This study proves that the “repeated donation” game is a good protocol to model and test cooperative behavior in laboratory rats, offering the opportunity to explore the neurobiology of cooperation.

In our lab, we are currently developing some novel paradigms to identify the empathy-driven change in prosocial behavior. We developed an experimental setting whereby rats learn to push a syringe to let a food pellet drop. Once they have been trained to push in order to obtain food for themselves, the test phase is consisting of dividing the arena (by means of a grid) into two separate halves: in this way, any one rat of a dyad will be able, by pushing, to let the food pellet drop towards the other conspecific and vice versa. In such way, the both of them can eat only by reciprocal pushing, while if only one subject of a dyad pushes, only the other will eat (Festucci et al., 2019).

We hereby propose that the exact rate at which the subjects will push, within a given session, may well depend on the status of the conspecific found behind the dividing grid. Our goal is to demonstrate that such prosocial habit is sensitive to the level of e.g., stress, or hunger, of the recipient. We propose that such a modulation of the prosocial habit, if demonstrated, would imply a metacognitive step: if the donor increases donations when the recipient is stressed or hungry, then the recipient is felt by the donor as a “subject” that may experience stress or hunger.

## Conclusions

Today, hundreds of research articles are available which studied the neural mechanism underlying human empathy. Most of the work focused on a detailed characterization of two processes: a *mirroring of the emotional response* i.e., living “as if the same feelings or perceptions occurred to me” and a *mentalization* as a cognitive evaluation of the others’ internal state (thoughts, intentions, feelings, perceptions).

Classic works, developed from 1995 to 2000, have gathered fundamental knowledge of the neural activity underlying these two processes, who seem to be completely separated at least at first glance. Current psycho-biological models of empathy nowadays consider the two processes deeply interactive, along with prosocial motivation.

Neuroscientists have often adopted consolidated concepts used by psychologists to describe empathic processes: sharing of emotion, role taking, Theory of Mind. Some researchers define empathy referring to the multi-dimensional construct, which comprehends its dual nature, both cognitive and affective. Other researchers refer to empathy taking into account only one component, either affective (studies concerning contact, pain and smell) or cognitive (Völlm et al., 2006). Some other authors suggest that, even though cognitive empathy seems to be depending on deductive abilities also supporting Theory of Mind, it is not equivalent to Theory of Mind. Good cognitive empathic skills are likely to rely on the same underlying skills that also enable mentalization, yet cognitive empathy is concerned with the attribution of emotions as opposed to cognitions; as such, the two constructs are potentially dissociable (Reniers et al., 2011).

Some studies (Lombardo et al., 2009; Schnell et al., 2011) surprisingly demonstrate that cognitive empathy leads to a “simulated” generation of affective states through the activation of the limbic system in the absence of external emotive signals.

Additional ambiguity comes from the definition of cognitive empathy vs. affective subcomponents of Theory of Mind. Both are often similarly defined in terms of comprehension of emotions. Shamay–Tsoory is the first author who tried to clarify the situation, elaborating a model in which a specific role was defined for all of the components. Albeit pitfalls encountered in the study of empathy may be troublesome, such kind of problems naturally came to surface during initial studies of any complex psychological phenomenon. In neuroscience, simplified stimuli and tasks have been fundamental to track the roots of empathic sub-processes, and such localization was absolutely necessary to continue with the study. Social-cognitive naturalistic paradigms are becoming frequent in neurosciences and some of them have already evidenced limitations of previous visions of empathy that focused on a single process. Such researches have shown that neural systems involved with experience-based sharing and mentalization, other than being activated in separate ways, are normally co-activated when the person is subject to complex social signals.

Moreover, functional neuroimaging will not have to be the sole research approach to study biological substrates of empathy: important outcomes could well come from genetics (Ebstein et al., 2010), from pharmacology (Bartz et al., 2011), from clinical studies on patients (Shamay-Tsoory and Aharon-Peretz, 2007) and from many other techniques. Finally, new approaches will not have to be limited to the study of healthy population. For example, naturalistic studies (Courchesne and Pierce, 2005) are demonstrating that people diagnosed with autism can be identified by difficulty in integrating multiple social signals: such an anomaly may be related to interruptions of connections between neural systems. Such widening of views will allow to plan for novel interventions with specific therapies, addressed towards children, teenagers and adults with deficiencies in multiple aspects of empathy and mentalization.

## Author Contributions

LC and LB wrote a first draft of this review. MC and FF added specific sections. SLR translated the first draft into English. RT and WA commented on the first draft and extended it. SC coordinated the whole workgroup.

## Conflict of Interest Statement

The authors declare that the research was conducted in the absence of any commercial or financial relationships that could be construed as a potential conflict of interest.

## References

[B1] Abraham E. Abraham E. Hendler T. Zagoory-Sharon O. Feldman R. (2016). Network integrity of the parental brain in infancy supports the development of children’s social competencies. Soc. Cogn. Affect. Neurosci. 11, 1707–1718. 10.1093/scan/nsw090 27369068 PMC5091682

[B2] Abraham E. Raz G. Zagoory-Sharon O. Feldman R. (2018). Empathy networks in the parental brain and their long-term effects on children’s stress reactivity and behavior adaptation. Neuropsychologia 116, 75–85. 10.1016/j.neuropsychologia.2017.04.015 28412510

[B3] Acevedo B. P. Poulin M. J. Brown L. L. (2019). Beyond romance: neural and genetic correlates of altruism in pair-bonds. Behav. Neurosci. 133, 18–31. 10.1037/bne0000293 30688485

[B4] Adam E. K. Gunnar M. R. (2001). Relationship functioning and home and work demands predict individual differences in diurnal cortisol patterns in women. Psychoneuroendocrinology 26, 189–208. 10.1016/s0306-4530(00)00045-7 11087964

[B5] Allman J. M. Tetreault N. A. Hakeem A. Y. Manaye K. F. Semendeferi K. Erwin J. M.. (2011). The von Economo neurons in the frontoinsular and anterior cingulate cortex. Ann. N Y Acad. Sci. 1225, 59–71. 10.1111/j.1749-6632.2011.06011.x 21534993 PMC3140770

[B6] Allport G. W. (1937). The personalistic psychology of William Stern. J. Pers. 5, 231–246. 10.1111/j.1467-6494.1937.tb02222.x

[B7] Ammaniti M. Lucarelli L. Cimino S. D’Olimpio F. (2004). Intergenerational transmission: feeding disorders in children and maternal psychopathology. Devenir 16, 173–198. 10.3917/dev.043.0173

[B8] Báez-Mendoza R. Schultz W. (2013). The role of the striatum in social behavior. Front. Neurosci. 7:233. 10.3389/fnins.2013.00233 24339801 PMC3857563

[B9] Baron-Cohen S. (2011). Zero Degrees of Empathy: A New Theory of Human Cruelty. London: Penguin.

[B10] Baron-Cohen S. Knickmeyer R. C. Belmonte M. K. (2005). Sex differences in the brain: implications for explaining autism. Science 310, 819–823. 10.1126/science.1115455 16272115

[B11] Baron-Cohen S. O’Riordan M. Jones R. Stone V. Plaisted K. (1999). A new test of social sensitivity: detection of faux pas in normal children and children with Asperger syndrome. J. Autism Dev. Dis. 29, 407–418. 10.1023/A:1023035012436 10587887

[B12] Baron-Cohen S. Wheelwright S. Hill J. Raste Y. Plumb I. (2001). The “reading the mind in the eyes” test revised version: a study with normal adults and adults with Asperger syndrome or high-functioning autism. Front. Psychiatry 42, 241–251. 10.1017/s0021963001006643 11280420

[B13] Barraza J. A. Zak P. J. (2009). Empathy toward strangers triggers oxytocin release and subsequent generosity. Ann. N Y Acad. Sci. 1167, 182–189. 10.1111/j.1749-6632.2009.04504.x 19580564

[B14] Bartolomeo L. Cerniglia L. Capobianco M. (2018). Empatia e Teoria della Mente: una review narrativa su differenze e convergenze concettuali alla luce delle recenti scoperte neurobiologiche. Ricerche di Psicologia 41, 19–54. 10.3280/RIP2018-001003

[B15] Bartz J. A. Zaki J. Bolger N. Ochsner K. N. (2011). Social effects of oxytocin in humans: context and person matter. Trends Cogn. Sci. 15, 301–309. 10.1016/j.tics.2011.05.002 21696997

[B16] Batson C. D. Batson J. G. Slingsby J. K. Harrell K. L. Peekna H. M. Todd R. M. (1991). Empathic joy and the empathy-altruism hypothesis. J. Pers. Soc. Psychol. 61, 413–426. 10.1037/0022-3514.61.3.413 1941512

[B17] Ben-Ami Bartal I. Decety J. Mason P. (2011). Empathy and prosocial behavior in rats. Science 334, 1427–1430. 10.1126/science.1210789 22158823 PMC3760221

[B19] Billeci L. Muratori P. Calderoni S. Chericoni N. Levantini V. Milone A.. (2019). Emotional processing deficits in Italian children with Disruptive Behavior Disorder: the role of callous unemotional traits. Behav. Res. Ther. 113, 32–38. 10.1016/j.brat.2018.12.011 30590200

[B20] Bischof-Köhler D. (1991). “The development of empathy in infants,” in Infant Development: Perspectives from German-Speaking Countries, eds Lamb M. E. Keller H. (Hillsdale, NJ: Lawrence Erlbaum), 245–273.

[B21] Blair J. Sellars C. Strickland I. Clark F. Williams A. Smith M. (1996). Theory of mind in the psychopath. J. Forensic. Psychiatry 7, 15–25. 10.1080/09585189608409914

[B24] Booth A. Granger D. A. Shirtcliff E. A. (2008). Gender-and age-related differences in the association between social relationship quality and trait levels of salivary cortisol. J. Res. Adolesc. 18, 239–260. 10.1111/j.1532-7795.2008.00559.x

[B25] Bora E. Gökçen S. Veznedaroglu B. (2008). Empathic abilities in people with schizophrenia. Psychiatry Res. 160, 23–29. 10.1016/j.psychres.2007.05.017 18514324

[B26] Botterill G. (1996). “Folk psychology and theoretical status,” in Theories of Theories of Mind, eds Carruthers P. Smith P. K. (Cambridge, MA: Cambridge University Press), 105–118.

[B27] Braaten E. B. Rosén L. A. (2000). Self-regulation of affect in attention deficit-hyperactivity disorder (ADHD) and non-ADHD boys: differences in empathic responding. J. Consult. Clin. Psychol. 68, 313–321. 10.1037/0022-006X.68.2.313 10780132

[B28] Bruner J. Feldman C. (1993). “Theories of mind and the problem of autism,” in Understanding Other Minds: Perspectives from Children with Autism, eds Baron-Cohen S. Tager-Flusberg H. Cohen D. (Oxford, UK: Oxford University Press), 267–291.

[B29] Buades-Rotger M. Beyer F. Krämer U. M. (2017). Avoidant responses to interpersonal provocation are associated with increased amygdala and decreased mentalizing network activity. eNeuro 4:ENEURO.0337-16.2017. 10.1523/eneuro.0337-16.2017 28660251 PMC5485378

[B30] Buchanan R. W. Kreyenbuhl J. Kelly D. L. Noel J. M. Boggs D. L. Fischer B. A. (2012). The 2009 schizophrenia PORT psychopharmacological treatment recommendations and summary statements. Focus 10, 194–216. 10.1176/appi.focus.10.2.194 PMC2800144 19955390

[B31] Cale E. M. Lilienfeld S. O. (2002). Sex differences in psychopathy and antisocial personality disorder: a review and integration. Clin. Psychol. Rev. 22, 1179–1207. 10.1016/s0272-7358(01)00125-8 12436810

[B32] Capobianco M. Cerniglia L. (2017). Early language development in preterm children without neurological damage: a longitudinal study. F1000Res. 6:2169. 10.12688/f1000research.13314.1 29445449 PMC5791006

[B34] Capobianco M. Cerniglia L. (2018a). Communicative, Cognitive and emotional issues in selective nutism: a narrative review on elements of a multimodal intervention. Interact. Stud. 19, 445–458. 10.1075/is.17018.cap

[B35] Capobianco M. Cerniglia L. (2018b). Evaluation strategies and cognitive intervention: the case of a monovular twin child affected by selective mutism. F1000Res. 7:221. 10.12688/f1000research.14014.1 29568498 PMC5840613

[B36] Capobianco M. Pizzuto E. A. Devescovi A. (2017). Gesture-speech combinations and early verbal abilities: new longitudinal data in the second year of age. Interact. Stud. 18, 54–76. 10.1075/is.18.1.03cap

[B38] Capps L. Kasari C. Yirmiya N. Sigman M. (1993). Parental perception of emotional expressiveness in children with autism. J. Consult. Clin. Psychol. 61, 475–484. 10.1037/0022-006x.61.3.475 8326050

[B39] Carnevali L. Montano N. Statello R. Coudè G. Vacondio F. Rivara S.. (2017). Social stress contagion in rats: behavioral, autonomic and neuroendocrine correlates. Psychoneuroendocrinology 82, 155–163. 10.1016/j.psyneuen.2017.05.017 28550792

[B40] Carr L. Iacoboni M. Dubeau M. C. Mazziotta J. C. Lenzi G. L. (2003). Neural mechanisms of empathy in humans: a relay from neural systems for imitation to limbic areas. Proc. Natl. Acad. Sci. U S A 100, 5497–5502. 10.1073/pnas.0935845100 12682281 PMC154373

[B42] Catmur C. (2015). Understanding intentions from actions: direct perception, inference, and the roles of mirror and mentalizing systems. Conscious. Cogn. 36, 426–433. 10.1016/j.concog.2015.03.012 25864592

[B43] Cerniglia L. Cimino S. Dentale F. Capobianco M. Tambelli R. (2018). Maternal symptioms of depression and paranoid ideation can be predictive of the onset of eating disoeders in early adolescents offspring: a nine year longitudinal study. Int. J. Psychol. Ther. 18, 221–234.

[B44] Chapman E. Baron-Cohen S. Auyeung B. Knickmeyer R. Taylor K. Hackett G. (2006). Fetal testosterone and empathy: evidence from the empathy quotient (EQ) and the “reading the mind in the eyes” test. Soc. Neurosci. 1, 135–148. 10.1080/17470910600992239 18633782

[B200] Cimino S. Cerniglia L. Porreca A. Ballarotto G. Marzilli E. Simonelli A. (2018). Impact of parental binge eating disorder: exploring children’s emotional/behavioral problems and the quality of parent-child feeding interactions. Infant Ment. Health J. 39, 552–568. 10.1002/imhj.21732 30084498

[B45] Clark T. F. Winkielman P. McIntosh D. N. (2008). Autism and the extraction of emotion from briefly presented facial expressions: stumbling at the first step of empathy. Emotion 8, 803–809. 10.1037/a0014124 19102591

[B46] Courchesne E. Pierce K. (2005). Why the frontal cortex in autism might be talking only to itself: local over-connectivity but long-distance disconnection. Curr. Opin. Neurobiol. 15, 225–230. 10.1016/j.conb.2005.03.001 15831407

[B47] Cuff B. M. Brown S. J. Taylor L. Howat D. J. (2016). Empathy: a review of the concept. Emot. Rev. 8, 144–153. 10.1177/1754073914558466

[B48] Cusi A. M. MacQueen G. M. Spreng R. N. McKinnon M. C. (2011). Altered empathic responding in major depressive disorder: relation to symptom severity, illness burden, and psychosocial outcome. Psychiatry Res. 188, 231–236. 10.1016/j.psychres.2011.04.013 21592584

[B49] Dadds M. R. Allen J. L. McGregor K. Woolgar M. Viding E. Scott S. (2014). Callous-unemotional traits in children and mechanisms of impaired eye contact during expressions of love: a treatment target? J. Child Psychol. Psychiatry 55, 771–780. 10.1111/jcpp.12155 24117894

[B50] Dadds M. R. Cauchi A. J. Wimalaweera S. Hawes D. J. Brennan J. (2012). Outcomes, moderators, and mediators of empathic-emotion recognition training for complex conduct problems in childhood. Psychiatry Res. 199, 201–207. 10.1016/j.psychres.2012.04.033 22703720

[B51] Dadds M. R. Hawes D. J. Frost A. D. Vassallo S. Bunn P. Hunter K.. (2009). Learning to ‘talk the talk’: the relationship of psychopathic traits to deficits in empathy across childhood. J. Child Psychol. Psychiatry 50, 599–606. 10.1111/j.1469-7610.2008.02058.x 19445007

[B52] Darwin C. (1872). The Origin of Species: By Means of Natural Selection Or the Preservation of Favored Races in the Struggle for Life (Vol. 1). New York, NY: Modern Library.

[B53] Davis M. H. (1980). Interpersonal Reactivity Index. Lewiston, NY: Edwin Mellen Press.

[B54] Davis M. H. (1983). The effects of dispositional empathy on emotional reactions and helping: a multidimensional approach. J. Pers. 51, 167–184. 10.1111/j.1467-6494.1983.tb00860.x

[B56] Decety J. E. Ickes W. E. (2009). The Social Neuroscience of Empathy. Cambridge, MA: MIT Press.

[B57] Derntl B. Finkelmeyer A. Eickhoff S. Kellermann T. Falkenberg D. I. Schneider F.. (2010). Multidimensional assessment of empathic abilities: neural correlates and gender differences. Psychoneuroendocrinology 35, 67–82. 10.1016/j.psyneuen.2009.10.006 19914001

[B58] Dolan M. Fullam R. (2004). Theory of mind and mentalizing ability in antisocial personality disorders with and without psychopathy. Psychol. Med. 34, 1093–1102. 10.1017/s0033291704002028 15554579

[B59] Dolivo V. Taborsky M. (2015). Norway rats reciprocate help according to the quality of help they received. Biol. Lett. 11:20140959. 10.1098/rsbl.2014.0959 25716088 PMC4360107

[B60] Domes G. Heinrichs M. Michel A. Berger C. Herpertz S. C. (2007). Oxytocin improves “mind-reading” in humans. Biol. Psychiatry 61, 731–733. 10.1016/j.biopsych.2006.07.015 17137561

[B61] Douglas H. M. Halverstadt B. A. Reinhart-Anez P. Webber E. S. Cromwell H. C. (2018). A possible social relative reward effect: influences of outcome inequity between rats during operant responding. Behav. Processes. 157, 459–469. 10.1016/j.beproc.2018.06.016 29990520

[B63] Dymond R. F. (1949). A scale for the measurement of empathic ability. J. Consult. Psychol. 13, 127–133. 10.1037/h0061728 18118267

[B65] Ebstein R. P. Israel S. Chew S. H. Zhong S. Knafo A. (2010). Genetics of human social behavior. Neuron 65, 831–844. 10.1016/j.neuron.2010.02.020 20346758

[B66] Egger M. Smith G. D. Altman D. G. (2001). Systematic Reviews in Health Care: Meta-Analysis in Context. London, UK: BMJ Publishing Group.

[B67] Eisenberg N. Miller P. A. (1987). The relation of empathy to prosocial and related behaviors. Psychol. Bull. 101, 91–119. 10.1037/0033-2909.101.1.91 3562705

[B68] Eres R. Decety J. Louis W. R. Molenberghs P. (2015). Individual differences in local gray matter density are associated with differences in affective and cognitive empathy. Neuroimage 117, 305–310. 10.1016/j.neuroimage.2015.05.038 26008886

[B69] Fadiga L. Craighero L. Olivier E. (2005). Human motor cortex excitability during the perception of others’ action. Curr. Opin. Neurobiol. 15, 213–218. 10.1016/j.conb.2005.03.013 15831405

[B71] Farrow T. F. Zheng Y. Wilkinson I. D. Spence S. A. Deakin J. W. Tarrier N.. (2001). Investigating the functional anatomy of empathy and forgiveness. Neuroreport 12, 2433–2438. 10.1097/00001756-200108080-00029 11496124

[B72] Feldman R. (2007). Mother-infant synchrony and the development of moral orientation in childhood and adolescence: direct and indirect mechanisms of developmental continuity. Am. J. Orthopsychiatry 77, 582–597. 10.1037/0002-9432.77.4.582 18194038

[B73] Feldman R. Zagoory-Sharon O. Weisman O. Schneiderman I. Gordon I. Maoz R.. (2012). Sensitive parenting is associated with plasma oxytocin and polymorphisms in the OXTR and CD38 genes. Biol. Psychiatry 72, 175–181. 10.1016/j.biopsych.2011.12.025 22336563

[B74] Ferrari R. (2015). Writing narrative-style literature reviews. Med. Writ. 24, 230–235. 10.1179/2047480615z.000000000329

[B75] Feshbach N. D. (1983). Learning to care: a positive approach to child training and discipline. J. Clin. Child Psychol. 12, 266–271. 10.1207/s15374424jccp1203_6

[B76] Festucci F. Buccheri C. Curcio G. Adriani W. (2019). “A new paradigm for Prosocial Behavior and Reciprocity, assessed in WT and HET rats for the DAT gene,” in Abstract to the “6th European Student Conference on Behaviour and Cognition”, Padova, Italy, 4–7. 10.1016/j.bbr.2020.112746 32502514

[B77] Fischer-Shofty M. Shamay-Tsoory S. G. Harari H. Levkovitz Y. (2010). The effect of intranasal administration of oxytocin on fear recognition. Neuropsychologia 48, 179–184. 10.1016/j.neuropsychologia.2009.09.003 19747930

[B78] Fletcher P. C. Happé F. Frith U. Baker S. C. Dolan R. J. Frackowiak R. S.. (1995). Other minds in the brain: a functional imaging study of “theory of mind” in story comprehension. Cognition 57, 109–128. 10.1016/0010-0277(95)00692-r 8556839

[B80] Fonagy P. Gergely G. Target M. (2007). The parent-infant dyad and the construction of the subjective self. J. Child Psychol. Psychiatry 48, 288–328. 10.1111/j.1469-7610.2007.01727.x 17355400

[B79] Fonagy P. Target M. (1996). Playing with reality: I. Theory of mind and the normal development of psychic reality. Int. J. Psychoanalysis 77, 217–233. 8771375

[B81] Frith C. D. Frith U. (2006). The neural basis of mentalizing. Neuron 50, 531–534. 10.1016/j.neuron.2006.05.001 16701204

[B82] Gallagher H. L. Frith C. D. (2003). Functional imaging of ‘theory of mind’. Trends Cogn. Sci. 7, 77–83. 10.1016/S1364-6613(02)00025-6 12584026

[B83] Gallagher H. L. Happé F. Brunswick N. Fletcher P. C. Frith U. Frith C. D. (2000). Reading the mind in cartoons and stories: an fMRI study of ‘theory of mind’in verbal and nonverbal tasks. Neuropsychologia 38, 11–21. 10.1016/s0028-3932(99)00053-6 10617288

[B85] Gallese V. (2001). The ‘shared manifold’ hypothesis. From mirror neurons to empathy. J. Conscious. Stud. 8, 5–7.

[B90] Gallese V. Fadiga L. Fogassi L. Rizzolatti G. (1996). Action recognition in the premotor cortex. Brain 119, 593–609. 10.1093/brain/119.2.593 8800951

[B84] Gallese V. Goldman A. (1998). Mirror neurons and the simulation theory of mind-reading. Trends Cogn. Sci. 2, 493–501. 10.1016/s1364-6613(98)01262-5 21227300

[B91] Gallese V. Keysers C. Rizzolatti G. (2004). A unifying view of the basis of social cognition. Trends Cogn. Sci. 8, 396–403. 10.1016/j.tics.2004.07.002 15350240

[B92] Glocker M. L. Langleben D. D. Ruparel K. Loughead J. W. Gur R. C. Sachser N. (2009). Baby schema in infant faces induces cuteness perception and motivation for caretaking in adults. Ethology 115, 257–263. 10.1111/j.1439-0310.2008.01603.x 22267884 PMC3260535

[B93] Goldman A. I. (1992). In defense of the simulation theory. Mind Lang. 7, 104–119. 10.1111/j.1468-0017.1992.tb00200.x

[B94] Gonzalez-Liencres C. Shamay-Tsoory S. G. Brüne M. (2013). Towards a neuroscience of empathy: ontogeny, phylogeny, brain mechanisms, context and psychopathology. Neurosci. Biobehav. Rev. 37, 1537–1548. 10.1016/j.neubiorev.2013.05.001 23680700

[B95] Gopnik A. (1996). “Theories and modules: creation myths, developmental realities and Neurath’s boat,” in Theories of Theories of Mind, Carruthers P. Smith P. K. (Cambridge, MA: Cambridge University Press), 169–183.

[B96] Grant M. J. Booth A. (2009). A typology of reviews: an analysis of 14 review types and associated methodologies. Health Info. Libr. J. 26, 91–108. 10.1111/j.1471-1842.2009.00848.x 19490148

[B97] Green B. N. Johnson C. D. Adams A. (2006). Writing narrative literature reviews for peer-reviewed journals: secrets of the trade. J. Chiropr. Med. 5, 101–117. 10.1016/s0899-3467(07)60142-6 19674681 PMC2647067

[B98] Haker H. Rössler W. (2009). Empathy in schizophrenia: impaired resonance. Eur. Arch. Psychiatry Clin. Neurosci. 259, 352–361. 10.1007/s00406-009-0007-3 19377866

[B99] Harari H. Shamay-Tsoory S. G. Ravid M. Levkovitz Y. (2010). Double dissociation between cognitive and affective empathy in borderline personality disorder. Psychiatry Res. 175, 277–279. 10.1016/j.psychres.2009.03.002 20045198

[B100] Harris P. (1996). “Desires, beliefs, and language,” in Theories of Theories of Mind, eds Carruthers P. Smith P. K. (Cambridge, MA: Cambridge University Press), 200–211.

[B101] Hastings P. D. McShane K. E. Parker R. Ladha F. (2007). Ready to make nice: parental socialization of young sons’ and daughters’ prosocial behaviors with peers. J. Genet. Psychol. 168, 177–200. 10.3200/gntp.168.2.177-200 17936971

[B201] Hernandez-Lallement J. van Wingerden M. Marx C. Srejic M. Kalenscher T. (2015). Rats prefer mutual rewards in a prosocial choice task. Front Neurosci. 8:443 10.3389/fnins.2014.00443 25642162 PMC4296215

[B102] Hoffman M. L. (1987). “The contribution of empathy to justice and moral judgment,” in Empathy and Its Development, eds Eisenberg N. Strayer J. (Cambridge, MA: Cambridge University Press), 47–80.

[B103] Hogan R. (1969). Development of an empathy scale. J. Consult. Clin. Psychol. 33, 307–316. 10.1037/h0027580 4389335

[B105] Ickes W. J. (Ed.). (1997). Empathic Accuracy. New York, NY: Guilford Press.

[B107] Isoda M. (2016). Understanding intentional actions from observers’ viewpoints: a social neuroscience perspective. Neurosci. Res. 112, 1–9. 10.1016/j.neures.2016.06.008 27393254

[B108] Jackson P. L. Meltzoff A. N. Decety J. (2005). How do we perceive the pain of others? A window into the neural processes involved in empathy. Neuroimage 24, 771–779. 10.1016/j.neuroimage.2004.09.006 15652312

[B111] Kalikow T. J. (1983). Konrad Lorenz’s ethological theory: explanation and ideology, 1938–1943. J. Hist. Biol. 16, 39–73. 10.1007/bf00186675 11611248

[B112] Kanske P. Böckler A. Trautwein F. M. Parianen Lesemann F. H. Singer T. (2016). Are strong empathizers better mentalizers? Evidence for independence and interaction between the routes of social cognition. Soc. Cogn. Affect. Neurosci. 11, 1383–1392. 10.1093/scan/nsw052 27129794 PMC5015801

[B114] Keum S. Shin H. S. (2016). Rodent models for studying empathy. Neurobiol. Learn. Mem. 135, 22–26. 10.1016/j.nlm.2016.07.022 27475995

[B115] Keysers C. Wicker B. Gazzola V. Anton J. L. Fogassi L. Gallese V. (2004). A touching sight: SII/PV activation during the observation and experience of touch. Neuron 42, 335–346. 10.1016/s0896-6273(04)00156-4 15091347

[B116] Knafo A. Plomin R. (2006). Prosocial behavior from early to middle childhood: genetic and environmental influences on stability and change. Dev. Psychol. 42, 771–786. 10.1037/0012-1649.42.5.771 16953685

[B117] Kohut H. (1984). How Does Analysis Cure? Chicago, IL: University of Chicago Press.

[B118] Laviola G. Zoratto F. Ingiosi D. Carito V. Huzard D. Fiore M.. (2017). Low empathy-like behavior in male mice associates with impaired sociability, emotional memory, physiological stress reactivity and variations in neurobiological regulations. PLoS One 12:e0188907. 10.1371/journal.pone.0188907 29200428 PMC5714342

[B119] Lenzi D. Trentini C. Pantano P. Macaluso E. Lenzi G. L. Ammaniti M. (2013). Attachment models affect brain responses in areas related to emotions and empathy in nulliparous women. Hum. Brain Mapp. 34, 1399–1414. 10.1002/hbm.21520 22359374 PMC6870475

[B120] Levy B. J. Wagner A. D. (2011). Cognitive control and right ventrolateral prefrontal cortex: reflexive reorienting, motor inhibition and action updating. Ann. N Y Acad. Sci. 1224, 40–62. 10.1111/j.1749-6632.2011.05958.x 21486295 PMC3079823

[B121] Li G. Wood R. I. (2017). Male rats play a repeated donation game. Physiol. Behav. 174, 95–103. 10.1016/j.physbeh.2017.03.010 28302575 PMC5420340

[B122] Lieberwirth C. Wang Z. (2016). The neurobiology of pair bond formation, bond disruption and social buffering. Curr. Opin. Neurobiol. 40, 8–13. 10.1016/j.conb.2016.05.006 27290660 PMC5072360

[B124] Lombardo M. V. Chakrabarti B. Baron-Cohen S. (2009). What neuroimaging and perceptions of self-other similarity can tell us about the mechanism underlying mentalizing. Behav. Brain Sci. 32, 152–153. 10.1017/s0140525x09000715

[B125] Luminet O. Grynberg D. Ruzette N. Mikolajczak M. (2011). Personality-dependent effects of oxytocin: greater social benefits for high alexithymia scorers. Biol. Psychol. 87, 401–406. 10.1016/j.biopsycho.2011.05.005 21641964

[B126] Luo S. Zhang T. Li W. Yu M. Hein G. Han S. (2019). Interactions between oxytocin receptor gene and intergroup relationship on empathic neural responses to others’ pain. Soc. Cogn. Affect. Neurosci. 14, 505–517. 10.1093/scan/nsz029 31070227 PMC6545534

[B127] Lutchmaya S. Baron-Cohen S. Raggatt P. (2002). Foetal testosterone and eye contact in 12-month-old human infants. Infant Behav. Dev. 25, 327–335. 10.1016/s0163-6383(02)00094-2

[B128] MacLean P. D. (1949). Psychosomatic disease and the “visceral brain”: recent developments bearing on the Papez theory of emotion. Psychosom. Med. 11, 338–353. 10.1097/00006842-194911000-00003 15410445

[B129] Mariscal M. G. Oztan O. Rose S. M. Libove R. A. Jackson L. P. Sumiyoshi R. D.. (2019). Blood oxytocin concentration positively predicts contagious yawning behavior in children with autism spectrum disorder. Autism Res. 12, 1156–1161. 10.1002/aur.2135 31132232 PMC6709875

[B130] Márquez C. Rennie S. M. Costa D. F. Moita M. A. (2015). Prosocial choice in rats depends on food-seeking behavior displayed by recipients. Curr. Biol. 25, 1736–1745. 10.1016/j.cub.2015.05.018 26051895

[B131] McIntosh D. N. Reichmann-Decker A. Winkielman P. Wilbarger J. L. (2006). When the social mirror breaks: deficits in automatic, but not voluntary, mimicry of emotional facial expressions in autism. Dev. Sci. 9, 295–302. 10.1111/j.1467-7687.2006.00492.x 16669800

[B132] Mead G. H. Mind H. (1934). Self and Society. Chicago, IL: University of Chicago.

[B135] Mier D. Lis S. Esslinger C. Sauer C. Hagenhoff M. Ulferts J.. (2013). Neuronal correlates of social cognition in borderline personality disorder. Soc. Cogn. Affect. Neurosci. 8, 531–537. 10.1093/scan/nss028 22362841 PMC3682436

[B136] Miklikowska M. Duriez B. Soenens B. (2011). Family roots of empathy-related characteristics: the role of perceived maternal and paternal need support in adolescence. Dev. Psychol. 47, 1342–1352. 10.1037/a0024726 21767014

[B137] Minio-Paluello I. Baron-Cohen S. Avenanti A. Walsh V. Aglioti S. M. (2009). Absence of embodied empathy during pain observation in Asperger syndrome. Biol. Psychiatry 65, 55–62. 10.1016/j.biopsych.2008.08.006 18814863

[B202] Mitchell J. P. Macrae C. N. Banaji M. R. (2005). Forming impressions of people versus inanimate objects: social-cognitive processing in the medial prefrontal cortex. Neuroimage 26, 251–257. 10.1016/j.neuroimage.2005.01.031 15862225

[B138] Mogil J. S. (2012). The surprising empathic abilities of rodents. Trends Cogn. Sci. 16, 143–144. 10.1016/j.tics.2011.12.012 22206750

[B139] Montoya F. G. Alcayde A. Baños R. Manzano-Agugliaro F. (2018). A fast method for identifying worldwide scientific collaborations using the Scopus database. Telematics Inform. 35, 168–185. 10.1016/j.tele.2017.10.010

[B140] Muratori F. Apicella F. Muratori P. Maestro S. (2011). Intersubjective disruptions and caregiver-infant interaction in early autistic disorder. Res. Autism Spectrum Dis. 5, 408–417. 10.1016/j.rasd.2010.06.003

[B141] Nakayama Y. Takahashi T. Wakabayashi A. Oono H. Radford M. H. (2007). Sex differences in the relationship between cortisol levels and the empathy and systemizing quotients in humans. Neuro Endocrinol. Lett. 28, 445–448. 10.1159/000072786 17693971

[B142] Novembre G. Zanon M. Silani G. (2015). Empathy for social exclusion involves the sensory-discriminative component of pain: a within-subject fMRI study. Soc. Cogn. Affect. Neurosci. 10, 153–164. 10.1093/scan/nsu038 24563529 PMC4321615

[B143] Osaka N. Osaka M. Morishita M. Kondo H. Fukuyama H. (2004). A word expressing affective pain activates the anterior cingulate cortex in the human brain: an fMRI study. Behav. Brain Res. 153, 123–127. 10.1016/j.bbr.2003.11.013 15219713

[B144] Pan L. (2008). Preparing Literature Reviews: Qualitative and Quantitative Approaches. 3rd Edn. Glendale, CA: Pyrczak Publishing.

[B145] Panksepp J. Panksepp J. B. (2013). Toward a cross-species understanding of empathy. Trends Neurosci. 36, 489–496. 10.1016/j.tins.2013.04.009 23746460 PMC3839944

[B146] Papez J. W. (1937). A proposed mechanism of emotion. Archiv. Neurol. Psychiatry 38, 725–743. 10.1001/archneurpsyc.1937.02260220069003

[B147] Pizzuto E. A. Capobianco M. (2008). Is pointing “just” pointing? Unraveling the complexity of indexes in spoken and signed discourse. Gesture 8, 82–103. 10.1075/gest.8.1.07piz

[B148] Premack D. Woodruff G. (1978). Does the chimpanzee have a theory of mind? Behav. Brain Sci. 1, 515–526. 10.1017/s0140525x00076512

[B149] Preston S. D. de Waal F. B. (2002). Empathy: its ultimate and proximate bases. Behav. Brain Sci. 25, 1–20. 10.1017/s0140525x02000018 12625087

[B151] Reniers R. L. Corcoran R. Drake R. Shryane N. M. Völlm B. A. (2011). The QCAE: a questionnaire of cognitive and affective empathy. J. Pers. Assess. 93, 84–95. 10.1080/00223891.2010.528484 21184334

[B152] Rizzolatti G. (2005). The mirror neuron system and its function in humans. Anat. Embryol. 210, 419–421. 10.1007/s00429-005-0039-z 16222545

[B153] Rizzolatti G. Craighero L. (2004). The mirror-neuron system. Annu. Rev. Neurosci. 27, 169–192. 10.1146/annurev.neuro.27.070203.144230 15217330

[B155] Rizzolatti G. Fadiga L. Gallese V. Fogassi L. (1996). Premotor cortex and the recognition of motor actions. Cogn. Brain Res. 3, 131–141. 10.1016/0926-6410(95)00038-0 8713554

[B154] Rizzolatti G. Sinigaglia C. (2010). The functional role of the parieto-frontal mirror circuit: interpretations and misinterpretations. Nat. Rev. Neurosci. 11, 264–274. 10.1038/nrn2805 20216547

[B156] Roche Olivar R. (2002). L’intelligenza Prosociale. Trento: Erikson.

[B157] Rogers C. R. (1959). Significant learning in therapy and in education. Educational Leadership 16, 232–242.

[B158] Ruby P. Decety J. (2003). What you believe versus what you think they believe: a neuroimaging study of conceptual perspective-taking. Eur. J. Neurosci. 17, 2475–2480. 10.1046/j.1460-9568.2003.02673.x 12814380

[B159] Rutte C. Taborsky M. (2007). Generalized reciprocity in rats. PLoS Biol. 5:e196. 10.1371/journal.pbio.0050196 17608566 PMC1914408

[B160] Sato N. Tan L. Tate K. Okada M. (2015). Rats demonstrate helping behavior toward a soaked conspecific. Anim. Cogn. 18, 1039–1047. 10.1007/s10071-015-0872-2 25964095

[B161] Schnell K. Bluschke S. Konradt B. Walter H. (2011). Functional relations of empathy and mentalizing: an fMRI study on the neural basis of cognitive empathy. Neuroimage 54, 1743–1754. 10.1016/j.neuroimage.2010.08.024 20728556

[B162] Schulze L. Lischke A. Greif J. Herpertz S. C. Heinrichs M. Domes G. (2011). Oxytocin increases recognition of masked emotional faces. Psychoneuroendocrinology 36, 1378–1382. 10.1016/j.psyneuen.2011.03.011 21477929

[B163] Schwenck C. Schmitt D. Sievers S. Romanos M. Warnke A. Schneider W. (2011). Cognitive and emotional empathy in children with ADHD and conduct disorder. Z. Kinder Jugendpsychiatr. Psychother. 39, 265–276. 10.1024/1422-4917/a000118 21667451

[B164] Sebastian C. L. Fontaine N. M. Bird G. Blakemore S. J. De Brito S. A. McCrory E. J.. (2011). Neural processing associated with cognitive and affective Theory of Mind in adolescents and adults. Soc. Cogn. Affect. Neurosci. 7, 53–63. 10.1093/scan/nsr023 21467048 PMC3252629

[B166] Shamay-Tsoory S. G. Aharon-Peretz J. (2007). Dissociable prefrontal networks for cognitive and affective theory of mind: a lesion study. Neuropsychologia 45, 3054–3067. 10.1016/j.neuropsychologia.2007.05.021 17640690

[B167] Shamay-Tsoory S. G. Aharon-Peretz J. Perry D. (2009). Two systems for empathy: a double dissociation between emotional and cognitive empathy in inferior frontal gyrus versus ventromedial prefrontal lesions. Brain 132, 617–627. 10.1093/brain/awn279 18971202

[B168] Shamay-Tsoory S. G. Harari H. Aharon-Peretz J. Levkovitz Y. (2010). The role of the orbitofrontal cortex in affective theory of mind deficits in criminal offenders with psychopathic tendencies. Cortex 46, 668–677. 10.1016/j.cortex.2009.04.008 19501818

[B165] Shamay-Tsoory S. G. Tomer R. Yaniv S. Aharon-Peretz J. (2002). Empathy deficits in Asperger syndrome: a cognitive profile. Neurocase 8, 245–252. 10.1093/neucas/8.3.245 12119321

[B171] Silberberg A. Allouch C. Sandfort S. Kearns D. Karpel H. Slotnick B. (2014). Desire for social contact, not empathy, may explain “rescue” behavior in rats. Anim. Cogn. 17, 609–618. 10.1007/s10071-013-0692-1 24126919

[B173] Singer T. (2006). The neuronal basis and ontogeny of empathy and mind reading: review of literature and implications for future research. Neurosci. Biobehav. Rev. 30, 855–863. 10.1016/j.neubiorev.2006.06.011 16904182

[B172] Singer T. Fehr E. (2005). The neuroeconomics of mind reading and empathy. Am. Econ. Rev. 95, 340–345. 10.1257/000282805774670103 29125271

[B174] Singer T. Seymour B. O’Doherty J. Kaube H. Dolan R. J. Frith C. D. (2004). Empathy for pain involves the affective but not sensory components of pain. Science 303, 1157–1162. 10.1126/science.1093535 14976305

[B175] Smith A. (2006). Cognitive empathy and emotional empathy in human behavior and evolution. Psychol. Record 56, 3–21. 10.1007/bf03395534

[B176] Smith A. (2009). The empathy imbalance hypothesis of autism: a theoretical approach to cognitive and emotional empathy in autistic development. Psychol. Record 59, 489–510. 10.1007/bf03395675

[B177] Soenens B. Berzonsky M. D. Papini D. R. (2016). Attending to the role of identity exploration in self-esteem: longitudinal associations between identity styles and two features of self-esteem. Intl. J. Behav. Dev. 40, 420–430. 10.1177/0165025415602560

[B178] Sperduti M. Guionnet S. Fossati P. Nadel J. (2014). Mirror neuron system and mentalizing system connect during online social interaction. Cogn. Process. 15, 307–316. 10.1007/s10339-014-0600-x 24414614

[B181] Stallings J. Fleming A. S. Corter C. Worthman C. Steiner M. (2001). The effects of infant cries and odors on sympathy, cortisol and autonomic responses in new mothers and non-postpartum women. Parenting 1, 71–100. 10.1207/s15327922par011&2_5

[B182] Stern D. N. (1977). The First Relationship: Mother and Infant. Cambridge, MA: Harvard University Press.

[B183] Tambelli R. Cerniglia L. Cimino S. Ballarotto G. (2015). Parent-infant interactions in families with women diagnosed with postnatal depression: a longitudinal study on the effects of a psychodynamic treatment. Front. Psychol. 6:2010. 10.3389/fpsyg.2015.01210 26322008 PMC4531209

[B184] Tennes K. Kreye M. (1985). Children’s adrenocortical responses to classroom activities and tests in elementary school. Psychosom. Med. 47, 451–460. 10.1097/00006842-198509000-00005 4059479

[B185] Terburg D. Morgan B. van Honk J. (2009). The testosterone-cortisol ratio: a hormonal marker for proneness to social aggression. Intl. J. Law Psychiatry 32:216. 10.1016/j.ijlp.2009.04.008 19446881

[B186] Tidoni E. Candidi M. (2016). Commentary: understanding intentions from actions: direct perception, inference and the roles of mirror and mentalizing systems. Front. Behav. Neurosci. 10:13. 10.3389/fnbeh.2016.00013 26903829 PMC4749676

[B187] Tomasello M. (1999). The human adaptation for culture. Annual. Rev. Anthropol. 28, 509–529. 10.1146/annurev.anthro.28.1.509

[B188] Valk S. L. Bernhardt B. C. Bockler A. Trautwein F. M. Kanske P. Singer T. (2016). Socio-cognitive phenotypes differentially modulate large-scale structural covariance networks. Cereb. Cortex 27, 1358–1368. 10.1093/cercor/bhv319 26733538

[B189] Van Overwalle F. Baetens K. (2009). Understanding others’ actions and goals by the mirror and mentalizing systems: a meta-analysis. Neuroimage 48, 564–584. 10.1016/j.neuroimage.2009.06.009 19524046

[B190] Völlm B. A. Taylor A. N. Richardson P. Corcoran R. Stirling J. McKie S.. (2006). Neuronal correlates of theory of mind and empathy: a functional magnetic resonance imaging study in a nonverbal task. Neuroimage 29, 90–98. 10.1016/j.neuroimage.2005.07.022 16122944

[B191] Wicker B. Keysers C. Plailly J. Royet J. P. Gallese V. Rizzolatti G. (2003). Both of us disgusted in my insula: the common neural basis of seeing and feeling disgust. Neuron 40, 655–664. 10.1016/s0896-6273(03)00679-2 14642287

[B192] Wimmer H. Perner J. (1983). Beliefs about beliefs: representation and constraining function of wrong beliefs in young children’s understanding of deception. Cognition 13, 103–128. 10.1016/0010-0277(83)90004-5 6681741

[B194] Xi C. Zhu Y. Niu C. Zhu C. Lee T. M. Tian Y.. (2011). Contributions of subregions of the pre-frontal cortex to the theory of mind and decision making. Behav. Brain Res. 221, 587–593. 10.1016/j.bbr.2010.09.031 20934455

[B196] Yildirim B. O. Derksen J. J. (2012). A review on the relationship between testosterone and life-course persistent antisocial behavior. Psychiatry Res. 200, 984–1010. 10.1016/j.psychres.2012.07.044 22925371

[B197] Zaki J. Ochsner K. N. (2012). The neuroscience of empathy: progress, pitfalls and promise. Nat. Neurosci. 15, 675–680. 10.1038/nn.3085 22504346

